# Using a Respectful Approach to Child-centred Healthcare (ReACH) in a paediatric clinical trial: A feasibility study

**DOI:** 10.1371/journal.pone.0241764

**Published:** 2020-11-09

**Authors:** Analise Nicholl, Kate Evelegh, Kane Evan Deering, Kate Russell, David Lawrence, Philippa Lyons-Wall, Therese Anne O’Sullivan

**Affiliations:** 1 School of Medical and Health Sciences, Edith Cowan University, Joondalup, Perth, Western Australia, Australia; 2 Peaceful Parents, Confident Kids, Toowoomba, Queensland, Australia; 3 Faculty of Arts, Business, Law and Education, Graduate School of Education, University of Western Australia, Perth, Western Australia, Australia; Pennington Biomedical Research Center, UNITED STATES

## Abstract

**Background:**

There is a growing momentum in paediatric ethics to develop respectful research and healthcare protocols. We developed, tested and refined our ‘Respectful Approach to Child-centred Healthcare’ (ReACH), to underpin respectful participant interactions in a clinical trial.

**Objective:**

To determine whether a ReACH-based approach is acceptable to children and parents, and effective in obtaining compliance with common healthcare assessments in a clinical trial of healthy 4-6-year-old children.

**Methods:**

ReACH-based child assessments were evaluated at two baseline clinics and one post-intervention, using mixed methods. Children (n = 49; 46.9% female; mean age = 5.24±0.88 years at baseline) and their parents provided independent evaluation, via customised 5-point Likert scales and qualitative feedback. A dedicated child researcher evaluated adherence to the study ReACH principles.

**Results:**

Children achieved compliance rates of 95% for body composition (BodPod) assessments; 89% for blood pressure measurements, and 92% (baseline) and 87% (post-intervention) for blood draws. Adherence to ReACH principles during clinic visits was positively associated with child compliance, significantly for baseline BodPod (p = 0.002) and blood test (p = 0.009) clinics. Satisfaction with BodPod protocols was positively associated with compliance, for children at baseline (p = 0.029) and for parents post-intervention (p <0.001). Parents rated the study itself very highly, with 91.7% satisfied at baseline and 100% post-intervention. Qualitative feedback reflected an enjoyable study experience for both parents and children.

**Conclusions:**

Adherence to our emerging ReACH approach was associated with high child compliance rates for common healthcare assessments, although no causality can be inferred at this preliminary stage of development. Participants expressed satisfaction with all aspects of the study. Our use of child-centred methods throughout a research intervention appears feasible and acceptable to children and their parents.

## Introduction

There is a growing momentum in paediatric ethics to develop respectful research and healthcare protocols, based on the rights of the child. Children are a vulnerable population, entitled to protection from unnecessary medical procedures, fear and pain, according to the United Nations Convention on the Rights of the Child [1]. However, societal concerns and parental emotional involvement can act to delay or prevent certain types of paediatric research [2], effectively denying sick children their entitlement to access evidence-based treatment [1].

According to the Declaration of Helsinki, all population groups should have equal access to appropriate participation in medical research [3]. However, treatments for children have often been adapted from those for adults, with limited testing [4] for their safety, efficacy or even acceptability to children, in effect creating ‘therapeutic and pharmaceutical orphans’ [5, 6]. In the USA, the National Institutes of Health prioritise child participation in clinical trials, as the developing bodies and brains of children respond to medicines and treatments differently to adults [7]. The field of child health needs more successful and well-designed clinical trials to grow the paediatric evidence-base [8]. Hence, the USA and Europe have legislated that children must be included in clinical and pharmacological trials [9, 10].

Despite legislation and growing international consensus that it is vital to include children of all ages in research [11], application has been limited. This may be in part because it has traditionally been easier and more economical to apply adult knowledge and treatments to children as though they were small adults [12]. Ethical issues such as questionable assent, physical and/or mental risk and discomfort can be particularly compromising for young children, especially if they do not directly benefit from the research [13–16].

While due care needs to be given to children’s preferences in healthcare [8], depending on their competence [17], there are currently few practical solutions in the literature for optimising the child’s experience during common assessments. Of these, blood tests and needle procedures, in particular, can have lasting negative effects on children, with on-going consequences such as increased anxiety, distress and reduced healthcare follow up [18, 19]. Fear of needles or injections (trypanophobia) has been variously reported as 22–75% of the paediatric population, depending on age, health and frequency of needle-procedures [20]; needle fear can adversely affect both children and parents [20, 21], as we found in our own pre-trial community consultation [22]. Children can feel intimidated by unfamiliar faces as well as settings [23].

Child dissent is another factor that has to be considered. Dissent can be assumed if, for example, a child’s behaviour varies substantially from normal during a health procedure: this can include non-verbal cues such as exhibiting passivity, or refusing to make eye-contact with a healthcare worker or researcher [4, 24, 25]. Such workers may not be trained to recognise all signals of child distress in anticipation of, or participation in, such a procedure, and hence may not regard this as genuine dissent [15]. Overriding a child’s expressed preferences can affect their behaviour, and could result in psychological harm, but harm is less likely when the concept of dissent is considered in risk/benefit calculations [24].

Another ethical issue encountered in paediatric research is obtaining informed assent from the child, which differs from the informed consent legally required from parents. When the child gives an affirmative response, as opposed to failing to object, it is referred to as ‘assent’–asking for a formal signature is not required [24]. The ethical issue of appropriate developmental age for such assent is also controversial [7]. Currently, consent must be given on a child’s behalf by at least one legal guardian [3]. Such ‘surrogate’ or ‘proxy’ parental consent is challenging, as parents trying to act in the best interests of their children often worry their decisions may harm or disadvantage their child [2, 13, 26, 27], which can affect objective decision-making.

In practice and in policy, healthcare settings can benefit from active and respectful consideration of children’s preferences. However, there is limited information on how to include this kind of approach in research and healthcare protocols. We found little current evidence of community or stakeholder consultation [28–30], of plans to maintain participant engagement [31], and of qualitative research to improve clinical trial design [32, 33], particularly in the paediatric setting.

Nursing models of care, such as patient-, family- and child-centred care, appeared a promising basis for improved child participation in the healthcare setting; however, relevant application of such models in specific paediatric research or healthcare settings was relatively rare at the time of our study, with translation from theory to practice noted to have unintentional consequences, such as providing barriers to child or family participation in the care process [17, 25, 30, 34, 35].

Although family-centred models of care operate with collaboration between health care professionals and parents [36], the focus can move away from the patient, and the child can be excluded from even a limited input into their own care [17]. Some children have expressed feeling marginalised by health professionals, researchers and their parents, noting in healthcare settings that these adults may talk around them, rather than to them, or offer ‘choices’ that are then disregarded [30, 34]. Such difficulties and controversies experienced in family-centred care have led to preferences for a more inclusive focus on the child as being central to the family unit of care: this emerging field, focus, framework or model is increasingly referred to as child-centred care [17, 25, 36].

As our preferred approach of child-centred care was more theoretical than practical, or even a model, when we began our research, we investigated a respectful model of child-centred care used in the practical child-care setting. Resources for Infant Educarers (RIE)^TM^ was developed by child-care specialist Magda Gerber for parents and families of very young children in day-care, based on the work of Hungarian paediatrician Emmi Pikler in improving the care of institutionalised children by childcare professionals [37–39]. This approach places emphasis on the caregiver developing a cooperative relationship with the child, rather than using authority or incentives. Three underlying principles appeared particularly relevant for child health interventions: using authentic communication, acknowledging emotions and inviting participation.

We consulted a childcare specialist who uses and teaches practical applications of this approach, to inform development of research strategies and assessments grounded in these principles. Our aim was to find practical ways to address several theoretical aspirations, such as how to validate the research experience for the child, and to provide a research environment conducive to play to help reduce anxiety and assist the child to feel in control [22, 23, 39]. With proper application, a child-focused approach could lead to increased acceptance of health assessments by children, improved research participation rates and greater interest in health and science from a young age. In light of current trends and legislation in paediatric ethics, improving child research methods could also help increase traditionally low rates of child participation in trials and increase their access to evidence-based healthcare [16].

We used the above considerations to develop, test and refine our own paediatric model, a Respectful Approach to Child-centred Healthcare (ReACH). In seeking to maintain the child participant as the centre of our focus, while allowing for full participatory partnership with their parents [17]—or child-centred care with positive aspects of family-centred care [35]—our model can be seen to be strongly influenced by Bronfenbrenner’s ecological model of child development [40, 41]. This model places the developing child at the centre of a nested range of settings, from the immediate family setting, through interactions with direct and indirect layers of family, social and cultural settings, and also considers the way changes in these larger settings over time can influence the developing child.

Our design of child-centred assessments also considered findings from our community consultation with parents [22] in developing appropriate procedures and resources for our clinical trial. We aimed to examine whether incorporating our emerging ReACH approach into a paediatric clinical trial would prove acceptable to child participants and their parents, with the secondary outcome of whether clinic ReACH adherence could be associated with child compliance with common health-related assessments. We believe our results from this feasibility study give preliminary support to the value of using a child-centred approach, but the approach needs further refinement and testing, in both the research and healthcare settings.

## Materials and methods

**Fig 1:**
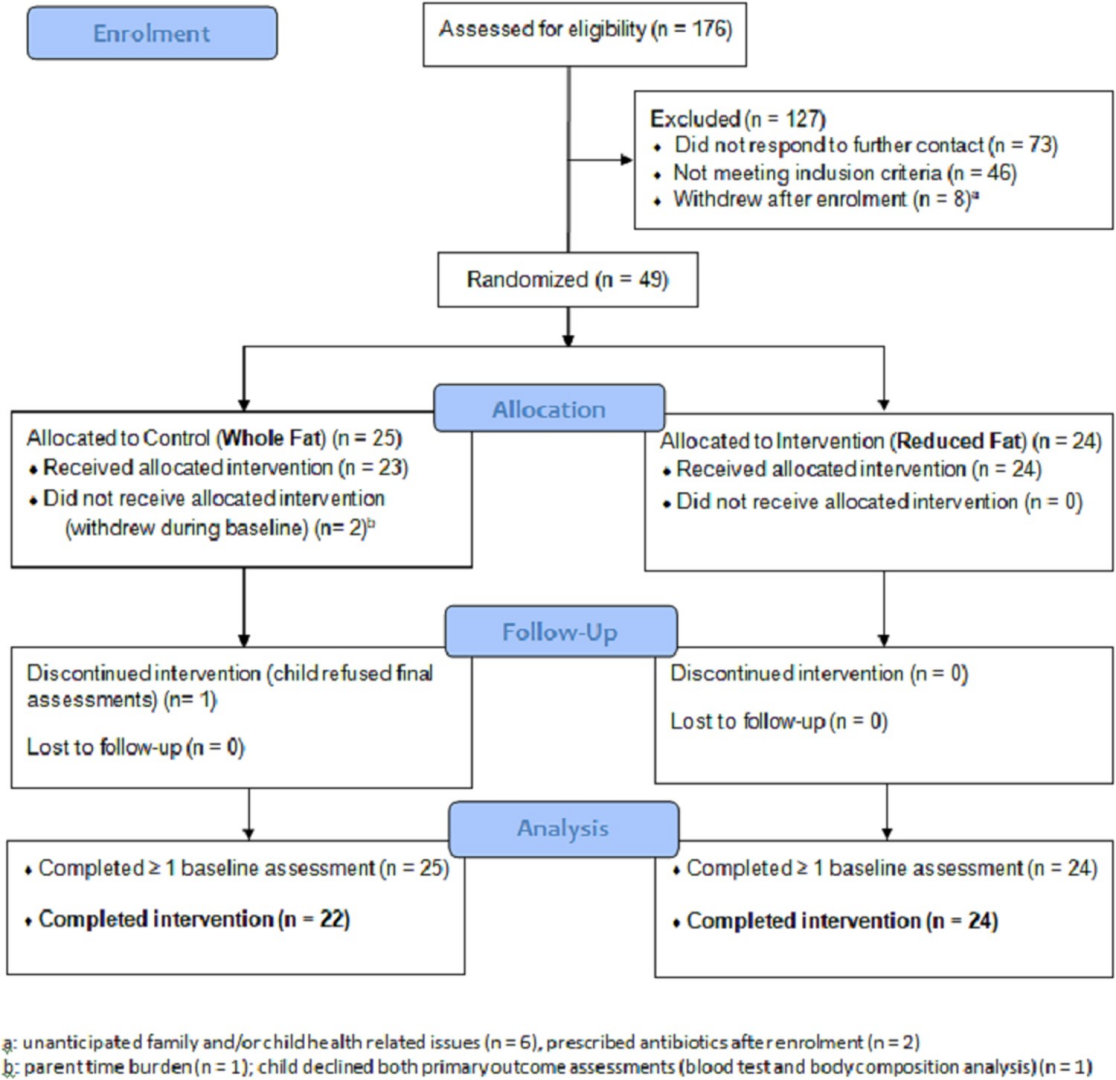
Flow diagram for the Milky Way Study, detailing study participant recruitment, randomisation and retention pre- and post-intervention (intervention duration = three months).

The Milky Way Study is a double-blind randomised controlled trial (RCT) investigating the effects of dairy fat on cardiometabolic and gut health in young West Australian children. It was prospectively registered with the Australia New Zealand Clinical Trials Registry as ACTRN12616001642471 [https://www.anzctr.org.au/Trial/Registration/TrialReview.aspx?id=371803]. The clinical trial involved clinical assessments at two baseline clinic visits, a dairy intervention of three months and repeat assessments at one final clinic visit; follow-up procedures three months later were home-based. The Milky Way Study was approved by the Edith Cowan University (ECU) Human Research Ethics Committee (Project no. 14990), and any specific participant details or images included in this publication have complied with the following provision: ‘the individual in this manuscript, or their legal guardian, has given written informed consent (as outlined in PLOS consent form) to publish these case details’. Recruitment took place over the first nine months of 2017; all Milky Way Study clinic visits were held from January-December 2017. The CONSORT flow diagram for the clinical trial is represented in **Fig 1**.

### Subjects

Healthy children aged 4–6 years were eligible for recruitment into the Milky Way Study if they were daily consumers of whole fat dairy products, living within 20 km of the ECU Joondalup campus and able to attend three clinic visits on campus. Children were excluded for recent antibiotic use and having a body weight lower than 9.5 kg: guidelines for total blood volume that can safely be taken from children stipulate age-related body weights [42]. Forty-nine children met these criteria and were enrolled as participants in the clinical trial.

### Study design

We used a mixed methods study design to evaluate our Respectful Approach to Child-centred Healthcare (ReACH) over all Milky Way Study clinic visits. A ReACH researcher rated adherence to the approach at each session. Participants and their parents were asked to provide independent evaluations of each clinic visit, scored via customised 5-point Likert scales. Qualitative feedback was recorded from structured discussions with children, open-ended questions in parent surveys and discussions with parents at study clinic visits.

The United Nations Convention on the Rights of the Child [1] has been a key influence on models of paediatric participation and care [17, 25, 35], including development of a Council of Europe model for child participation and associated Child Participation Assessment Tool [43]. Child-centred care appeared to offer us an improved focus on the child compared with a specifically family-centred model of care [17, 35, 36]. Initial development of our ReACH approach involved consultation with a childcare specialist on the practical application of three key principles from the Gerber-Pikler RIE^TM^ approach, as used and taught in the childcare setting [37–39]. These were incorporated into the Milky Way Study clinical trial protocol as an aspirational base for applying a respectful child-centred approach throughout all child interactions and assessments. Development, application, evaluation and refinement of our ReACH model show strong influences of the Bronfenbrenner ecological model of child development [40], in which the perceptions and interactions of the developing child can be seen as affected by a range of environmental settings, including the family ecology, and their changes over time [41].

### Recruitment

Parents who expressed interest in enrolling their child(ren) in the study were contacted by telephone to discuss the study in further detail. We designed all recruitment materials in accordance with the study ReACH principles. Study ReACH resources emailed to all enrolled families included information and education aimed both at the level of the child and of caregivers:

- A Parent Information Leaflet and a copy of the Informed Consent form, to complement previous information, answer early questions and avoid study process misunderstandings [22, 27, 44]
- A pictorial Child Information Leaflet, featuring all assessments, as part of our age-appropriate informed assent process [11, 45]
- A link to a five-minute video on the Milky Way Study Facebook page [https://www.facebook.com/milkywaystudy/videos/402125510141202/], which used a guided tour to provide information about each clinic visit [45], including child discussion after procedures. This kind of approach has been recommended by both children and parents to inform multimedia websites that help children decide about clinical trial recruitment [28].

These materials helped prepare children to meet their child-focused clinic ReACH researcher and the research team; provided visual details of assessment rooms and equipment, and pointed out their personal contribution to research.

### Informed consent and assent

To build familiarity and establish trust, we had the same two-person team of a child-focused ReACH researcher and a lead researcher present at all clinic visits with the same family wherever possible [16]. The rationale behind having the same ReACH child researcher present was to build and maintain a trusting relationship with each child, maintain child focus during all assessments and to advocate for them at all times. Caregivers [hereafter referred to as ‘parents’, as there was always at least one attending parent at clinics] were given time to ask questions before signing informed consent.

Australian ethical guidelines consider 4–6 year-olds too limited in understanding and capacity for discussion to give meaningful assent [7, 24]. We investigated whether application of our respectful approach might provide a pathway to informed assent by our participants, within the bounds of our study.

### Incorporating ReACH principles into the Milky Way Study

Our ReACH philosophy and principles, as developed and refined over study clinical assessments, have been summarised in **Box 1**. Contributing sources are discussed and cited in the text, as appropriate.

**Box 1. A Respectful Approach to Child-centred Healthcare (ReACH) principles and philosophy**

**Philosophy**

- Children have the same rights and needs to participate in research as adults, but with all the protections from exploitation that should be afforded any vulnerable group
- Children have the right to direct informed assent to procedures and risks, to the extent of their developmental maturity rather than a specific age
- Parents/caregivers are always to be respected as an authority on their own child

**Principles**

- Use authentic communication
- Acknowledge emotions
- Invite participation

**Application**

- Appropriate information and education should be provided in advance at the level of both the child and the caregivers
- Avoid distracting children from understanding what is happening, or offering them incentives to participate in assessments
- Paediatric healthcare workers should be trained to recognise verbal and non-verbal dissent, and researchers, in particular, should not continue if the child remains unwilling after a suitable pause to explore their understanding of the procedure

**Recommendation**

- Healthcare and research institutions should have an appropriate, child-centred ethical model or code of practice that promotes these fundamental rights of the child

**ReACH mission statement**: Respect our young participants at all times

We embedded three key ReACH principles [38] into all our clinics:

1. Use authentic communication (honest explanations about why a test is needed, and what to expect, can empower the child).
2. Acknowledge emotions (acknowledge the child’s feelings are valid, including fear)
3. Invite participation (young children can participate by conducting a similar test on a teddy bear or toy, or by helping set up machines or equipment where suitable).

Each clinic was run by a lead researcher, known to the parent from recruitment. In many instances parents brought in older and/or younger siblings, affecting researcher focus in each case. While our primary focus at all clinics was our child participants, it was also important to maintain parent engagement over the length of a study with considerable parental burden. This included advocating for a balance between acknowledging familiar parenting behaviour and authority [18, 23, 46], and study ReACH principles.

We developed a ReACH strategy to try to maintain ‘confident momentum’ following child assent. We defined this as allowing for individual engagement and comfort with the research experience, but keeping the overall experience moving forward at a reassuring pace, to minimise doubt and maintain confidence in the researchers.

In line with recommendations from our pre-trial community consultation focus groups, the study had a space-themed approach [22]. This was based on the appearance of our BodPod (see equipment and assessment details below), and aimed to boost participant engagement [31] by increasing the sense of fun of the experience.

### Clinical assessments

**Fig 2:**
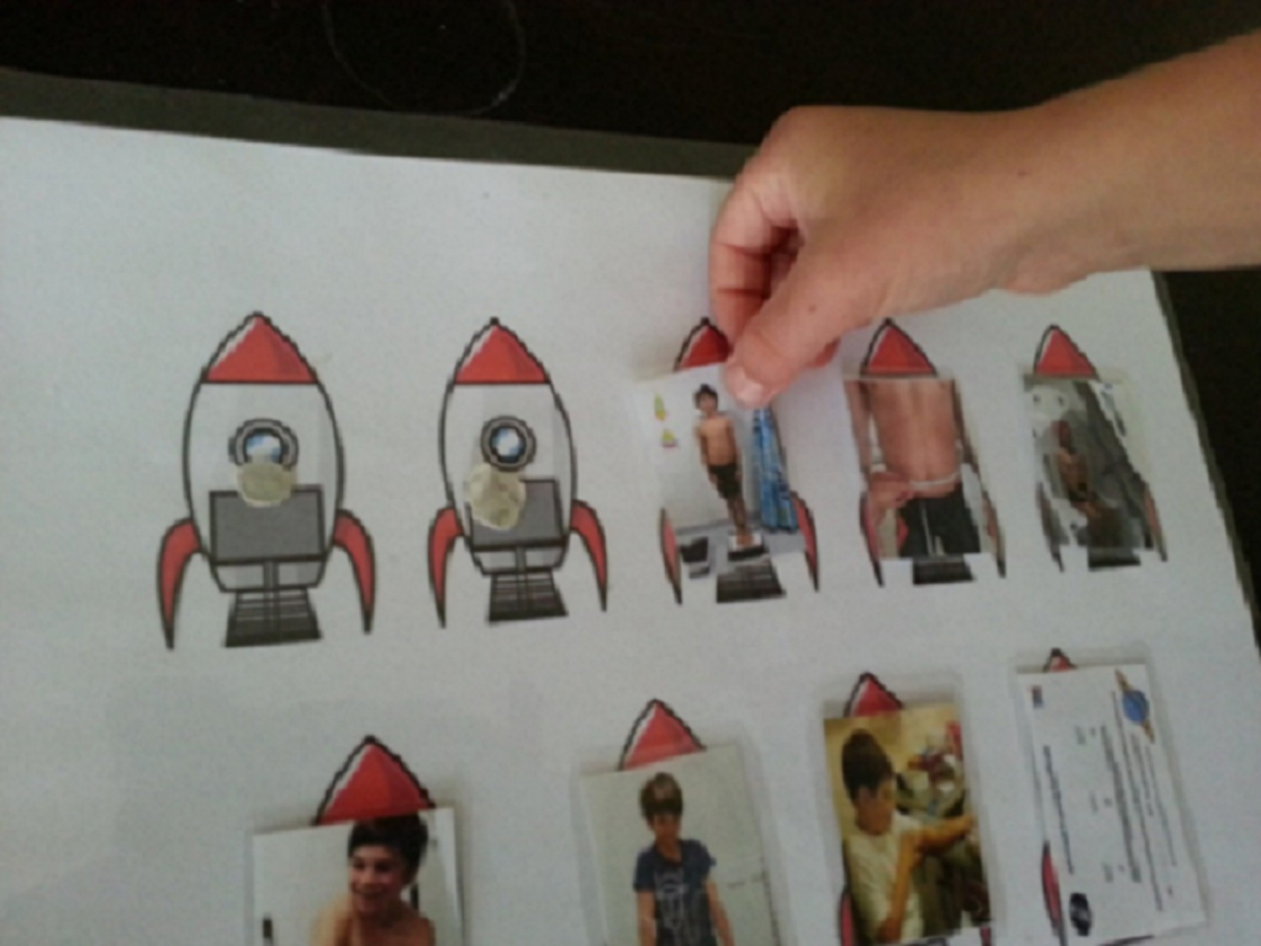
ReACH resource showing the sequence of tests at a trial clinic visit, with detachable photographs able to serve as a visual guide both to test procedures and to track progress over the visit: From left to right photographs present represent assessment of weight, waist measurement and BodPod body composition analysis (top row); blood pressure, strength, blood test and ‘graduation’ certificate awarded after all clinics completed (lower row, cut off). (Written informed consent has been provided by the legal guardian on behalf of the individual in these photographs (as outlined in PLOS consent form) to publish these case details. Fig 2 has been reprinted from [22] under a CC BY license, with permission from MDPI, original copyright 2018).

To allow adequate time for familiarity, we split baseline assessments over two clinic visits. We ran a single follow-up clinic, as children had already performed all the assessments at baseline and therefore did not need additional time for awareness and understanding. Fuller details of development and incorporation of ReACH principles into Milky Way Study procedures are given in **S2 File**. At the start of each clinic visit, the child and their ReACH researcher had around 15–20 minutes of ‘settle-in’ playtime. This allowed the ReACH researcher time for sensitive observation of the child, and allowed the child time for familiarisation with the clinic setting and the day’s assessments [23]. Each clinic had a pictorial schedule of assessments, a chart with detachable photographs that could be pulled off and posted in a special post-box when each assessment was completed, as shown in **Fig 2**. (The legal guardian of the participant in this figure has given written informed consent (as outlined in PLOS consent form) to publish these details). This acted as a timeline for the session. We considered our combination of pre-education, playtime, familiarisation and invited participation to be a respectful and appropriate level of informed assent.

#### Baseline body composition assessments

**Fig 3:**
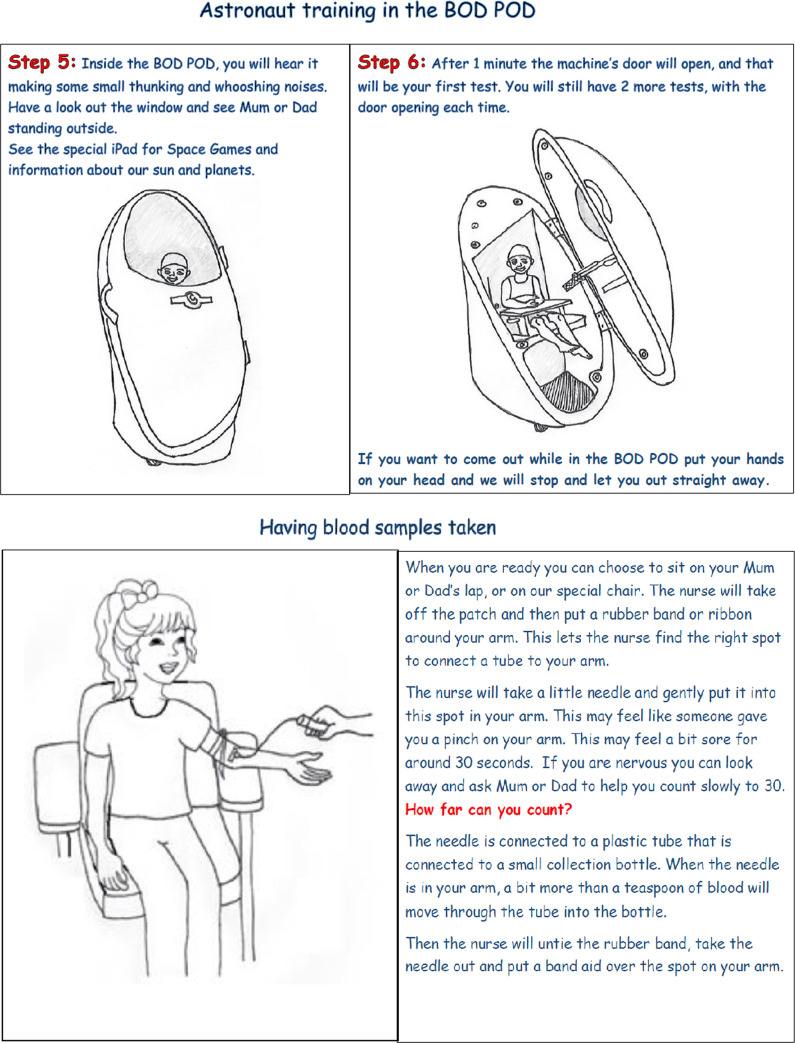
Excerpts from the Milky Way Study Child Information Leaflet: *Upper*: Astronaut training in the Bod Pod. The Bod Pod uses non-invasive air-displacement to measure body composition (lean mass and fat mass). Participants need to sit inside during testing, and there is a paediatric seat for children under six years of age. *Lower*: Having blood samples taken. (Pages reproduced from the Milky Way Study Child Information Leaflet. Fig 3 was previously published as two separate figures, and has been reprinted from [22] under a CC BY license, with permission from MDPI, original copyright 2018).

The first clinic visit involved collecting anthropometric measurements, including weight and height; neck and waist circumference, and determination of body fat mass and fat-free mass; we also determined blood pressure. Body composition was measured in the BodPod (COSMED, Rome, Italy), as indicated in **Fig 3**. The study BodPod had a paediatric option (BodPod Pediatric Option^TM^ GS model, COSMED USA Inc., San Francisco, CA), which included a seat for children less than 6 years.

Blood pressure measurements were taken in the BodPod after three to five minutes at rest, using a calibrated Dinamap ProCare 300 Monitor (GE Medical Systems, Tampa, Florida) with an appropriate paediatric cuff. We provided an authentic explanation for all procedures, with key examples provided in **Box 2**.

**Box 2. ReACH-based explanations and basic strategies used in clinical assessments**

**Table:** 

**Procedure / equipment**	**Typical explanation**
Topical anaesthetic (EMLA) cream/patches	This cream will make the skin feel numb. Numb means when you can’t feel much in that spot for a while.
Cream activation time: 60 minutes	It will take about an hour to work. There are lots of toys you can choose from to play with while we wait, or games we can play.
Phlebotomy room	We can all go over to the collection room. It has everything we need in the room. Your mum/dad will come too.
Patch removal	We have a special adhesive remover so that we can remove your patch comfortably. It will take a bit longer, but we have found other children are much happier with this remover as the patch is very sticky.
Tourniquet	This is a bit like a seatbelt for your arm. It is used to help find the best veins. I will try it on both your arms today but just so that I can see which will be the best arm today. It may feel a bit tight.
**Blood collection**	I am ready to take a small amount of blood from just under the skin. The cream will have made the area numb, but you might feel some pressure. Because of the numbing cream some children say they don’t feel anything at all; others say they feel a poke on their skin. Everyone is different: you can tell me how you feel it worked for you. Some children like to watch, and others don’t: it is up to you. Your job is to sit nice and still and breathe in through the nose and out through the mouth. This will help you be the boss of your own feelings.
Protective tape after blood collection	You have been very brave today. I just need to put a bit of pressure on the spot where the blood was collected. In a minute we can tape a little cotton wool on the spot, and you can take this off after about 15 minutes, or longer if you want. The blood will stop coming out, and your body will fix up the hole quickly.
**Blood pressure cuff and procedure**	This is an arm pressure [*as opposed to ‘blood pressure’*] cuff; it goes around your arm and gets quite tight. It is a bit like a floatie [*a flotation device for both arms*, *used in swimming pools to help keep young children afloat*], where the air blows into the floatie and (it) hugs around your arm. It doesn’t stay tight for long, and we can watch how the numbers go down on this monitor as it slowly returns to normal. The arm pressure tells us how strongly your heart is pumping blood to your arm.
**BodPod body composition assessment**	The BodPod, or rocket ship, measures what your body is made of. There are a couple of things you need to know about our rocket ship, or BodPod. There is a window that you can see us through, and we can see you. I will be watching, and you will hear me talking to you, letting you know how much time there is left. If at any time you want me to open the door, you can place your hands on your head, like this, and I will open the door. I would like you to sit nice and still inside the rocket ship, and we will close the door for one minute and then open it to see how you are. This will happen 3 times. You will hear a whooshing noise—sounds like a “whoosh, whoosh” [*similar to a bicycle pump pumping up a tyre*]—and you will also hear a clunking noise—like a “clunk…… clunk”. These sounds mean that the machine is working.

After assessments, a choice of breakfast items was offered to all children, and the participant chose a small thank-you gift from a selection of items. The child was not aware of the thank you gift beforehand, to avoid this acting as an undue incentive.

#### Baseline blood test

The second baseline clinic visit required a fasting blood test. Application of anaesthetic patches is recommended to prevent child needle fear [21, 23, 47], and this was endorsed by parents at our pre-trial community consultation [22]. However, we considered it respectful to give children an informed choice. EMLA (AstraZeneca, Sweden) numbing patches were optimally applied to both inner arms, to allow the phlebotomist a choice of the best venipuncture site. Package instructions and our own in-house tests established that a 50-60-minute wait was needed for local cutaneous absorption and optimal numbness. A potential disadvantage was that the EMLA active ingredients, lidocaine and prilocaine, may have a side-effect of vein-shrinkage.

After children complained about painful removals of the anaesthetic patches, we added an adhesive-removal solvent wipe to the process (Smith & Nephew REMOVE Adhesive remover, St-Laurent, Canada. Ingredients: isoparaffin, dipropylene glycol methyl ether, aloe extract, benzyl alcohol, fragrance). The EMLA anaesthetic patches were largely effective at eliminating pain, but not feelings of touch or needle insertion. We made this distinction clear to the children and their parents prior to the test [23].

We employed an experienced paediatric phlebotomist, following our community consultation finding that most parents considered this the best guarantee of a good child experience [22]. By minimising both physical and psychological harm, and keeping the child and their parent informed and involved in the moment, we hoped that the children would feel they were doing something worthwhile even if it meant some discomfort [15]. In order to empower children who expressed fear about the test, we acknowledged their feelings and reassured them that the ReACH researcher had a strategy previously used to help other study children, placing them in charge of their own feelings. Children were not held down, as was the current accepted practice in paediatric phlebotomy at the time of writing [23, 48], but given the option of sitting with their parent if they preferred (as described in Fig 3). Our ReACH strategy for blood tests hence incorporated a combination of pharmacological and psychological approaches, ongoing monitoring of the participant and invited participation of the child and their parent in the way we implemented the blood test [49].

#### Final body composition and blood test assessments

The third clinic, held after three months of dairy intervention, incorporated all previous assessments. As per baseline procedures, we sent text and email reminders to help prepare the child and their family, and reviewed the day’s procedures at the start of the clinic [50], using the pictorial schedule shown in Fig 2.

### Additional ReACH strategies and tools

#### Enablers and barriers

Parents offering their children incentives to participate in assessments is ethically dubious. It may cause some children to believe there is something to fear about doing the assessments, thereby increasing the child’s anxiety and reducing the likelihood they will complete the assessment. Paediatric healthcare workers should be trained to recognise verbal and non-verbal dissent [15], and researchers, in particular, should not continue if the child remains clearly unwilling after a suitable pause to explore their understanding of the procedure [24]. Practical incorporation of ReACH principles into child assessments made us expand our ideas of incentives as ‘bribes’ or ‘distractions’, concepts often used interchangeably in the literature [23, 24], to incorporate the behavioural concepts of positive and negative reinforcement [51]. These were investigated for impact on our ReACH-based assessments.

We defined a bribe as a parent offering a form of positive reinforcement, such as a treat or reward for later (delayed gratification). Similarly, a distraction was defined as a parent offering any form of negative reinforcement that could break eye-contact with the researcher, trust and/or session momentum. Our ReACH approach was designed with the belief that distracting a child before they are fully ready for a procedure can result in a sudden awareness, such as finding a needle in their arm without warning, which could negate choice, validation of the experience and informed assent.

Study confident momentum tools included the age-appropriate explanations and basic strategies shown in Box 2. An iPad mini electronic touch screen tablet, programmed with space-themed and educational games, was initially used with the first 7% of children to undergo baseline assessments, particularly in the BodPod as the rocket ship ‘on-board computer’, to help keep children relaxed during the assessment [22, 52], but it proved too distracting.

#### ReACH Adherence Tool

The ReACH researcher assessed adherence to the child-centred approach during each clinic visit. Seven key principles were used to develop the tool’s adherence categories for our study: mutual trust, respect, sensitive observation, quality care-giving, a prepared environment, time for uninterrupted play and consistency [23, 39]. The ReACH Adherence Tool comprises 13 questions, as shown in **Box 3**. Application enabled the ReACH researcher to maintain a consistent clinical schedule, while tailoring each clinic around the specific characteristics of the child [45].

**Box 3. ReACH Adherence Tool for the child researcher to evaluate adherence to ReACH principles and the study protocol during each clinic visit. A score of 13 implies very high adherence**

**A Respectful Approach to Child-centred Healthcare (ReACH) Adherence Checklist**

For application by ReACH Researcher at each child-centred clinic visit

**TRUST**

1. **Researcher’s trust in child’s competence to complete session using**
  **ReACH approach only** Confident (1) Little/ no trust (0)
  Parent and child anxiety levels gauged on arrival and acknowledged by providing more time for introductions / play / session information as required
2. **Distraction from parent** None (1) ≥ 1 distraction (0)
  *E*.*g*. *‘Don’t look’ when child is distressed/ offers video clip on mobile phone / holds child’s head to look away*
3. **Bribe from parent** None (1) ≥ 1 distraction (0)
  *E*.*g*. *If you do this ‘I’ll take you to (*restaurant*)’ / ‘I’ll buy you*…*’*
4. **Distraction from researcher** None (1) ≥ 1 distraction (0)

**AUTHENTICITY** (Communication with child is honest, upfront and genuine)

5 **Study information/ video shown to child before assessments** Yes (1) No (0)
  Procedures explained via video / easy and engaging visual tools
6 **Researcher honest about potential discomfort** Yes (1) No (0)
  *Explore visual and sensory explanations*, *e*.*g*. *‘Some children say they feel some pressure*, *others say they don’t feel anything at all*. *Would you like me to show you on my arm*?*’* [If yes, Researcher applies pressure with fingernail onto own arm.] *‘You can try it on your arm*, *and tell me how it feels’*
7 **Researcher uses authentic language and tone** Yes (1) No (0)
  *Speaks authentically and explains procedures using simple photographic/ visual devices and open discussion (no baby talk*, *forced games or other placating examples)*
8 **Protocol went according to plan, as initially described to child** Yes (1) No (0)
  *Where protocol deviates*, *Researcher stops*, *acknowledges and explains the discrepancy before seeking child’s approval to continue*

**OBSERVATION** (Sensitive observation for insight into child’s needs)

9 **Researcher focus** Yes (1) No (0)
  *Researcher watches for and acknowledges distress cues*, *particularly when the child looks to the Researcher during explanations and procedures; maintains eye contact throughout procedures where has promised to do so*
10 **Researcher finds insights into child’s personality, to reach them on their level and make connection(s)** Yes (1) No (0)
  *During play time each child can form a unique connection with the Researcher*. *This develops and strengthens as the Researcher applies the ReACH approach*, *particularly if the child is interested in the research*

**ENVIRONMENT**

11 **Safe to express feelings** Feelings not investigated (0) Feelings investigated / acknowledged (1)
12 **Playtime allowed** <15 mins (0) ≥15 min (1)
13 **Control of play** (child instigates) Researcher led (0) Child led (1)

#### Child Comfort Evaluation Tool

**Fig 4:**
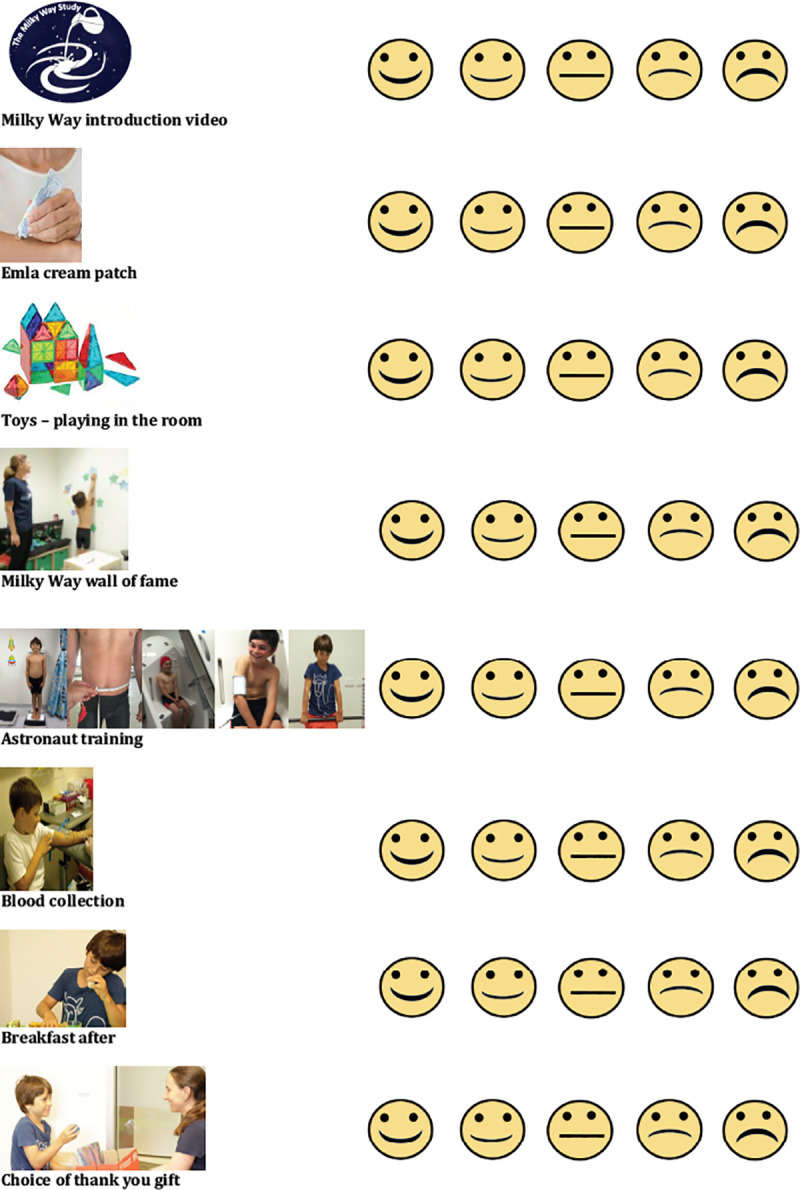
Child Comfort Evaluation Tool for self-evaluation of comfort and satisfaction with Milky Way Study clinic sessions and assessments. This figure is similar but not identical to the original image used in the study and is provided for illustrative purposes only. (Written informed consent has been provided by the legal guardian on behalf of the individual in these photographs (as outlined in PLOS consent form) to publish these case details).

The ReACH researcher developed a customised, laminated evaluation tool for each clinic visit, to assess child comfort (or satisfaction) with clinic procedures and assessments. This was derived from a combination of two tools validated for pre-assessment of dental anxiety in young children, the 7-faces Revised Smiley Faces Program, for computer application in children aged 4–11 years [53], and the recommended alternative for paper-based assessment, the 5-faces version of the Modified Child Dental Anxiety Scale, for children aged over five years [54]. Assessment and interpretation of pain in young children have traditionally been regarded as difficult [47], and there is as yet no validated scale for children less than six years to report acute pain, such as needle pain [55]. The study Child Comfort Evaluation Tool gave each child the opportunity to identify the appropriate face on a 5-faces Likert smiley-face assessment scale, placed opposite a colour photograph of each procedure in that clinic, as shown in **Fig 4** (the legal guardian of the participant in this figure has given written informed consent (as outlined in PLOS consent form) to publish these details).

### Evaluation of how we operationalized ReACH principles into practice

We attempted to evaluate the feasibility and appropriateness of the developing ReACH approach at both study time points. The ReACH researcher used the ReACH Adherence Tool (Box 3) to rate trust, authenticity, observation and environment via a binary response to each question: 1 (yes) or 0 (no). All scores were added to form a total adherence score, where a value closer to 13 implied highly satisfactory adherence to study ReACH principles.

Additionally, we asked both child and parent to provide feedback after each clinic visit, using the Child Comfort Evaluation Tool (Fig 4) and parental evaluation forms, making it clear to the children that their views and experiences were valued [11]. To overcome early response bias, or social desirability bias, we refined the child evaluation process as a quiet colouring-in activity after breakfast, with the child and their parent engaged at separate tables. Children appeared to rate each activity and assessment with due consideration, offering reasoned feedback and practical improvements to the ReACH researcher afterwards. Children rated their feelings about each of the appropriate clinic visit procedures under the following headings: 1) Study information; 2) Application of EMLA patches; 3) Toys and playtime; 4) Warm-up activity (decorating a star for the Milky Way Study ‘Wall of Fame’ opposite the BodPod); 5) ‘Astronaut Training’ BodPod and blood pressure assessments; 6) Blood test; 7) Breakfast choices afterwards, and 8) Choosing a gift. Child scores were assessed as big smile = 5 to big frown = 1, with a straight mouth/ undecided = 3.

Quantitative components of parent evaluations were based on responses via a customised 5-point Likert scale (5 = strongly agree to 1 = strongly disagree, with 3 = undecided). Qualitative evaluation included parents noting potential improvements, comparisons with previous blood tests and general comments. Questions used in the relevant evaluation forms are shown in **Box 4**. To reduce potential feedback bias, unidentified evaluation questionnaires were presented to parents in a plain envelope that was sealed after completion.

**Box 4. Parent evaluation forms for post-clinic assessment of Milky Way Study procedures and ReACH protocol (quantitative responses invited by ticking the appropriate response against a 5-point Likert scale (5 = strongly agree to 1 = strongly disagree, with 3 = undecided); qualitative responses to be written in the space provided)**

**BodPod Body Composition Initial and Final Clinics**

- Q1: The Research Team and pre-preparation helped me feel reasonably comfortable with my child’s blood test experience
- Q2: My child felt comfortable with the BodPod assessment
- Q3: Communication was honest and child-friendly
- Q4: I knew what was expected of me and my child
- Q5: My child was given sufficient opportunity to participate in the BodPod experience
- Q6: My child’s feelings were taken into consideration during testing
- Q7: We will be willing to consider another BodPod assessment in 3 months’ time, at study end OR We would recommend participation to other families
- Q8: Can you suggest any improvements we could make to BodPod Astronaut Training procedure that would make it a better experience for your child and/or you?

**Blood Test Initial and Final Clinics**

- Q1: The Research Team and pre-preparation helped me feel reasonably comfortable with my child’s blood test experience
- Q2: My child was willing to consider participation in the blood test [even if they didn’t go ahead]
- Q3: Communication was honest and child-friendly
- Q4: I knew what was expected of me and my child
- Q5: My child was adequately prepared for the blood sample
- Q6: My child was given sufficient opportunity to help in the set-up for the test
- Q7: My child’s feelings were taken into consideration during the testing
- Q8: We will be willing to consider another blood test in 3 months’ time, at study end [*Initial test only*]
- Q9: If your child has previously had a blood test, how did this blood test experience compare? OR If your child participated in the baseline blood test, how did this blood test experience compare?
- Q10: Can you suggest any improvements we could make to the blood collection procedure that would make it a better experience for your child and/or you?

**Milky Way Study Process after Initial and Final Clinics and Dairy Intervention**

- Q1: The study process overall so far has been acceptable to me
- Q2: The study process overall so far has been acceptable to my child
- Q3: I am willing to consider participating in these clinics again with my child, at study end [*initial clinic*] OR I would be willing to do this study again, or to recommend it to other parents [*final clinic*]
- Q4: I am willing to recommend the Milky Way Study to other parents [*Initial clinic only*]
- Q5: Can you suggest any improvements we could make to the study to make it a better experience for you and/or your child?
- Q6: If we wanted to ask a focus group of parents about ways to improve this study, would you be willing to be contacted to be part of this group, sometime in the next few years? [*Final clinic only*]

### Statistical analyses

IBM SPSS Statistics for Windows, Version 26.0 (Armonk, NY: IBM Corporation, released 2019) was used for all data analysis. As we did not use a control group as a comparison for our ReACH approach, we were not able to assess potential causality; however, we were interested in investigating whether our adherence to the ReACH-based procedures (as assessed by our adherence tool) was associated with subject compliance. Participant and family descriptive statistics have been presented as mean (± standard deviation) for continuous variables (all normally distributed), and as counts and percentages for frequencies. Child success and compliance rates with assessments were additionally stratified by sex.

The ReACH approach was applied to participants across each clinic visit; all clinics were assigned a score out of 13 using the ReACH Adherence Tool. Feedback and regular researcher discussion informed small process improvements; if successful, they became part of all subsequent clinic visits. Effects on outcomes due to familiarity with the Milky Way Study environment and procedures are suggested by changes in ReACH adherence scores and/or child compliance with tests as each child progressed through the study. At each time point we separately considered possible associations of ReACH adherence scores (all normally distributed) for those children who complied, and those who did not, using independent sample t-tests or one-way ANOVA, as appropriate.

Children evaluated procedural satisfaction with clinic assessments by marking the Child Comfort Evaluation Tool Likert faces scale, while parents separately used Likert scales to rate agreement with questions about the study child-centred procedures. Due to high overall evaluation ratings, we attempted to calculate comparable satisfaction scores using a binary cut-off classifications of 1 = satisfaction and 0 = qualified satisfaction/ uncertainty and/or a level of dissatisfaction, using a Likert score cut-off midway between agree (smile) and strongly agree (big smile). We used chi-squared tests to look for associations of child compliance with 1) parent and child satisfaction with assessments, and 2) parental offered bribes and/or distraction of their children during BodPod and blood tests. Cramer’s V was squared to obtain the effect size of significant associations.

Missing data, due to child withdrawal, instrument malfunction and blood test non-compliance are accounted for in table counts and footnotes. We requested evaluation of all clinics, key assessments and components, whether or not they were successful: we averaged parental scores to cover missing components for any clinics, and for children we consulted researcher clinic notes, to determine a suitable evaluation score for missing components from comments provided. However, where blood assessments were refused in advance, both children and parents did not evaluate that component of the clinic. A significance level of p < 0.05 was applied to all tests.

## Results

### Participant characteristics

Milky Way Study researchers recruited and randomised 49 children (46.9% female) into two dairy groups. All children participated in at least one set of clinic visit evaluations. Children ranged in age from 4.06 to 6.97 years at the first clinic, with a mean age of 5.24 (± 0.88) years: girls = 5.16 (± 0.77), boys = 5.31 (± 0.97) years. Three children (two girls) were withdrawn by their parents during the study (drop-out rate of 6%), due to refusal of key assessments (n = 1), perceived parent burden (n = 1) and child refusal to continue the dairy intervention (n = 1). **Table 1** shows child and parent characteristics and family sociodemographics.

**Table 1:** Milky Way Study participant baseline characteristics (n = 49).

Characteristics	n	Percentage/ Mean (± standard deviation)
Age (years)	49	5.24 ± 0.88
Female sex	23	46.9%
Number of siblings	47	
*0*	4	8.5%
*1*	23	48.9%
*2*	15	31.9%
*3*	5	10.6%
Mother education	46	
*Year 12 or less*	7	15.2%
*Trade*	0	0%
*Tertiary*	39	84.8%
Mother marital status	44	
*Defacto/Living with partner*	5	10.9%
*Divorced/Separated*	4	8.7%
*Married*	36	78.3%
*Single*	1	2.2%
Father education	46	
*Year 12 or less*	11	23.9%
*Trade*	4	8.7%
*Tertiary*	31	67.4%
Father marital status	46	
*Defacto/Living with partner*	4	8.7%
*Divorced/Separated*	5	10.9%
*Married*	36	78.3%
*Single*	1	2.2%
Participant time watching TV/ DVDs (hours/day)	41	1.38 ± 0.73
Participant time playing computer games (hours/day)	19	0.79 ± 0.45

### Participant compliance with clinical assessments

Assessment success and compliance rates for our three major assessments have been reported in **Table 2**. Child assessment success refers to successful completion of a clinical assessment. Child compliance refers to child preparedness to try the test on the day, even if no result was achieved due to unexpected circumstances, e.g. equipment failure, unsuitable veins. We have used child compliance rates in all evaluation analysis of our ReACH approach.

**Table 2:** Participant success and compliance rates for body composition (BodPod), blood pressure and blood samples at baseline and final clinic visits (three-month intervention). Compliance is regarded as being willing to attempt a clinic assessment on the day.

Clinic Assessment	Participants at clinic^a^			Assessment success			Assessment compliance		
	Total	Male	Female	Total	Male	Female	Total	Male	Female
**BodPod**^**b**^									
*Baseline*	49	26	23	46 (93.9%)	24	22	47 (95.9%)	25	22
*Final*	46	25	21	40 (87.0%)	21	19	44 (95.7%)	23	21
**Blood Pressure**^**c**^									
*Baseline*	49	26	23	39 (79.6%)	21	18	44 (89.8%)	24	20
*Final*	46	25	21	38 (82.6%)	19	19	41 (89.1%)	21	20
**Blood Test** ^**d**^^,^^**e**^^,^^**f**^									
*Baseline* ^**f**^	48 (1)	26	22 (1)	37 (77.1%)	22	15	44 (91.7%)	24	20
*Final*^**f**^	46 (3)	25 (1)	21 (2)	33 (71.7%)	18	15	40 (87.0%)	22	18

^**a**^Participant numbers present at clinics: n = 49 children were enrolled and attended the baseline BodPod and Blood Pressure clinic; n = 48 attended the baseline blood clinic (1 withdrawal from the study); n = 46 attended the final combined clinic (2 further withdrawals).

^**b**^BodPod malfunction at study mid-point (4 weeks awaiting repairs): we were unable to measure 2 boys at baseline and 5 children (3 girls) at the end of intervention; assessment success is reduced due to these numbers; they are considered compliant if they were willing to reschedule the test/expressed disappointment it was unavailable.

^**c**^Blood pressure: success is defined as ≥ 2 readings achieved; compliance as ≥ 1 reading achieved [optimally 3 readings attempted; the Dinamap ProCare 300 reinflates the cuff and rereads automatically, rejecting outliers, until each set of readings is within acceptable limits]. Blood pressure success rates were affected by the BodPod malfunction: as blood pressure was measured seated in the BodPod after 3–5 minutes at rest, a second change in protocol proved difficult for some children to adapt to.

^**d**^Blood test success and compliance: children were considered compliant if a failed test was rescheduled; if it proved successful, they were assessed as both compliant and successful; in two cases at baseline, however, a ReACH researcher was unable to be present at this second attempt, which prevented update of evaluation forms to reflect this success and the effects of the ReACH protocol; in two similar cases at final clinics a ReACH researcher was present to ensure appropriate updates.

^**e**^For the purposes of comparison with studies that recorded ‘first attempt’ success, or a successful initial venous access on the day: the 4 children referred to in **d** achieved a successful draw only at a second (follow-up) clinic, and 1 child each at baseline and final clinics achieved success via the second arm attempted. Successful first attempts are hence considered to be 34 (70.8%) at baseline and 30 (65.2%) at the final clinic.

^**f**^Blood test pre-clinic refusal: where the child and/or parent refused the blood test well in advance of the clinic, these children (1 girl at baseline and 3 children (2 girls) post-intervention) form a sub-category of non-compliance, pre-clinic refusal, as compared with non-compliance on the day; these numbers are indicated in parentheses, as these participants were present for the rest of the assessments scheduled for that clinic visit.

In a combined total of four cases (4.3% of the 94 participant attendances at all blood test clinics), the parent and/or child refused blood tests prior to the clinic (one child prior to both clinics, two children prior to the second clinic). These form a separate category of participant non-compliance, pre-clinic refusal, as the ReACH protocol was abbreviated and neither parent nor child evaluated this aspect of these clinics. Several blood tests were unsuccessful despite child compliance: in 10 cases (11.9% of 84 instances of compliance, or 10.6% of the 94 clinic attendances) the paediatric phlebotomist reported issues with unsuitable veins, the vein rolling or collapsing. In two cases (2.4% of total compliance) she subsequently achieved a successful draw from the second arm. Four children (4.8% of total compliance) refused initial or further attempts at a blood draw while in the phlebotomy room, and parents offered to bring them back for a second, successful attempt. In two of these cases at baseline the ReACH researcher was unable to be present, as these were rescheduled at the parent’s convenience; hence, clinic evaluation forms were not updated to reflect effects of the ReACH protocol on compliance, or satisfaction with this success.

### Evaluation of the ReACH approach and application in assessments

#### Researcher evaluation

Out of a maximum value of 13, the mean (± standard deviation) researcher-assessed ReACH adherence score from clinic visit one (baseline BodPod and blood pressure; n = 49 evaluated) was 10.16 ± 2.01(78.2% adherence); from clinic visit two (baseline blood test; n = 48 evaluated) the mean was 9.77 ± 2.30 (75.6% adherence), and from clinic visit three (all tests; n = 44 evaluated) the mean increased to 10.35 ± 1.48 (79.6% adherence).

**Table 3** shows that higher scores for clinic ReACH adherence were associated with higher compliance with assessments. A positive association between ReACH adherence and child compliance proved significant for baseline BodPod (p = 0.002) and baseline blood test (p = 0.009) clinics.

**Table 3:** Associations of clinic ReACH adherence scores (out of a maximum of 13) with child compliance for assessments at baseline and final clinics.

ReACH Score^a^	BodPod^b^^,^^c^			Blood Pressure^b^^,^^c^			Blood Test^b^^,^^c^		
(/13)	n	Mean ± SD	p	n	Mean ± SD	p	n	Mean ± SD	p
**Baseline clinic**	49	10.16 ± 2.01	0.002	49	10.16 ± 2.01	0.111	48	9.77 ± 2.30	0.009
*- Non-compliant*	2	6.00 ± 2.83		5	8.80 ± 2.95		3	6.33 ± 2.08	
*- Compliant*	47	10.34 ± 1.81		44	10.32 ± 1.86		44	9.91 ± 2.12	
*- Pre-clinic refusal* ^**d**^	0			0			1	13.00 ± 0.00	
**Final clinic**	46	10.35 ± 1.48	0.413	46	10.35 ± 1.48	0.131	46	10.35 ± 1.48	0.375
*- Non-compliant*	2	9.50 ± 0.71		5	9.40 ± 1.14		3	9.33 ± 0.58	
*- Compliant*	44	10.39 ± 1.50		41	10.46 ± 1.48		40	10.38 ± 1.55	
*- Pre-clinic refusal*^**d**^	0			0			3	11.00 ± 0.00	

SD: standard deviation

^**a**^The maximum ReACH adherence score achievable is 13, which implies very high adherence to the study child-centred approach.

^**b**^Study participant numbers present at clinics: n = 49 children were enrolled and attended the baseline BodPod clinic; n = 48 attended the baseline blood clinic (1 withdrawal from the study); n = 46 attended the final combined clinic (2 further withdrawals).

^**c**^Independent sample t-tests used for associations of BodPod and blood pressure adherence with compliance (2-tailed significance); one-way ANOVA used for blood tests (baseline Tukey post-hoc tests not performed with p<0.05 where any group had < 2 cases; no between-group significances at final clinic).

^**d**^Pre-clinic refusal: child and/or parent refused the blood test well in advance of the clinic (1 participant at baseline and 3 participants at final blood tests). These children all rated the overall clinic more highly than other participants.

#### Child and parent evaluation

Associations of child compliance with parent and child self-reported satisfaction at BodPod and blood test clinics are shown in **Table 4**. The majority of participants reported high levels of satisfaction with BodPod assessments at baseline (91.7% of children; 87.5% of parents) and final clinics (82.6% of children; 95.3% of parents), although five children (11%) were willing but unable to be assessed in the BodPod at the final clinic, due to equipment failure, which affected their clinic satisfaction ratings. Satisfaction was positively associated with compliance for children at the baseline BodPod assessment (p = 0.029), and for their parents at the final BodPod clinic (p < 0.001). Fewer children expressed satisfaction with blood tests at baseline (56.5% of children satisfied) and final clinics (58.1% satisfied) than their parents. Effect sizes were not considered meaningful owing to very low numbers of participants within certain groups in each analysis.

**Table 4:** Associations of parent and child self-reported satisfaction with child compliance at BodPod and blood test clinics, assessed using chi-squared tests and reported as numbers and percentages of the total.

Satisfaction^a^	Baseline Clinic^b^				Final Clinic^b^			
	Compliance				Compliance			
	Total^c^	Non-compliant	Compliant	p	Total^c^	Non-compliant	Compliant	p
BodPod								
Child	48	2 (4.2%)	46 (95.8%)	0.029	46	2 (4.3%)	44 (95.7%)	0.507
*- Less satisfied*	4 (8.3%)	1 (2.1%)	3 (6.3%)		8 (17.4%)	0 (0.0%)	8 (17.4%)	
*- Satisfied*	44 (91.7%)	1 (2.1%)	43 (89.6%)		38 (82.6%)	2 (4.3%)	36 (78.3%)	
Parent	48	2 (4.2%)	46 (95.8%)	0.101	43	2 (4.7%)	41 (95.3%)	<0.001
*- Less satisfied*	6 (12.5%)	1 (2.1%)	5 (10.4%)		2 (4.7%)	2 (4.7%)	0 (0.0%)	
*- Satisfied*	42 (87.5%)	1 (2.1%)	41 (85.4%)		41 (95.3%)	0 (0.0%)	41 (95.3%)	
**Blood test**^**d**^								
Child	46	2 (4.3%)	44 (95.7%)	0.099	43	3 (7.0%)	40 (93.0%)	0.756
*- Less satisfied*	20 (43.5%)	2 (4.3%)	18 (39.1%)		18 (41.9%)	1 (2.3%)	17 (39.5%)	
*- Satisfied*	26 (56.5%)	0 (0.0%)	26 (56.5%)		25 (58.1%)	2 (4.7%)	23 (53.5%)	
Parent	45	2 (4.4%)	43 (95.6%)	0.012	41	2 (4.9%)	39 (95.1%)	<0.001
*- Less satisfied*	3 (6.7%)	1 (2.2%)	2 (4.4%)		5 (12.2%)	2 (4.9%)	3 (7.3%)	
*- Satisfied*	42 (93.3%)	1 (2.2%)	41 (91.1%)		36 (87.8%)	0 (0.0%)	36 (87.8%)	

^**a**^Children evaluated their satisfaction by marking the pictorial study Child Comfort Tool, and parents separately used Likert scales to rate satisfaction with the Milky Way Study ReACH procedures, assessments and consideration of child participants. The ‘satisfied’ cut-off used for every assessment is midway between the Likert scale values of 4 = agree (smile/satisfied) and 5 = strongly agree (big smile/very satisfied), reflecting the high levels of satisfaction recorded; ‘less satisfied’, includes all levels of qualified satisfaction, uncertainty and dissatisfaction below the cut-off, including 4 = agree (smile), 3 = undecided, 2 = disagree (frown/unsatisfied) and 1 = strongly disagree (big frown/very unsatisfied).

^**b**^Study participant numbers present at clinics: n = 49 children were enrolled and attended the baseline BodPod clinic; n = 48 attended the baseline blood test clinic (1 withdrawal from the study); n = 46 attended the final combined clinic (2 further withdrawals).

^**c**^Total numbers reported here depend on child compliance with the assessment, and preparedness of the parent and the child to rate their satisfaction with the assessment itself if the child was not compliant.

^**d**^p = asymptotic significance, 2-sided.

^**e**^Where children refused blood tests in advance of the clinic, no satisfaction rating was recorded for the assessment by either the parent or the child. Pre-clinic blood test refusal numbers of one child at baseline and 3 children at final clinics have hence been omitted for this assessment analysis.

Parents rated the Milky Way Study itself very highly, with 44 (91.7%; n = 48 evaluations) satisfied at baseline, and 46 (100%) satisfied after the intervention and final clinic visit. Parents also provided informative and largely positive qualitative feedback relating to their child’s clinic visit and study experiences, with representative comments cited in **Box 5**.

**Box 5. Representative parent comments on Milky Way Study clinics, assessments and the study as a whole, at baseline and/or post-intervention**

**BodPod Body Composition Parent Feedback**

- *“Everything was great*! *Really made it easier on* [my child] *& myself*!*”*
- *“It was lovely*. *I really appreciated how* [my child] *was treated as someone who understood his body and study requirements rather than being treated as a baby”*
- *“*[My child] *was a little apprehensive after his first clinic but was able to go in the BodPod on the 2nd try & was very proud and happy once he had completed it”*
- *“It was great*! *I wish everything they do* [involving child health assessment] *was like that”*
- *“The team members were amazing—so understanding and great with both my kids* [participant and younger sibling]*”*
- “I think it was very well organised and executed”
- *“No improvements required as everything was clearly explained & my child was respected & made to feel comfortable*. *As a result*, *he enjoyed the BodPod experience”*

**Blood Tests**

- *“This was my child's first experience*, *and I couldn't ask for a better experience’*
- *“Much more positive* [than her previous experience with blood tests]. *The numbing cream helped lots as well as letting her help with setup”*
- *“It was great*. *My child enjoyed testing his blood”*
- *“Even better than the last time he had a blood collection*, *he was confident–well prepared due to the researcher”*
- *“Blood tests are difficult to do*. *My child had made up his mind from the start* [not to do it] *and he would not have changed his mind no matter what”*
- *“Better* [than her previous experience]: *greater care with explanations*, *more time for her to familiarise herself with the room*, *personnel and procedure”*
- *“His feelings*, *and fears*, *were fully taken into consideration*, *and previously the pain relief option wasn't given”*
- *“Better* [than previous experience] *because the children knew what to expect”*
- “I loved the breathing and empowering thinking”
- “New patches are much better than the first ones”
- *“The removal of the patch took longer but caused no pain as the researcher had explained*. *The patch removal was the only thing my child was concerned about”*
- *“The second time a better experience in terms of patch being removed*. *In terms of the actual blood draw both times were a good experience–definitely helped by the use of the EMLA”*
- *“This one was better*. *No pain*. *The new method of removing the EMLA patch worked well”*
- “*He was happier to do the baseline test*. *This time he was anxious and didn't want to go through with the test”*

**Milky Way Study after Initial and/or Final Clinics**

- *“I think it’s excellent*, *very friendly and appropriate”*
- *“You did a great job*, *and you covered all areas in a nice way”*
- *“All good*, *she was excited to come here again today”*
- “I found the study very easy to do and will be fascinated to see the results”
- *“It was already a good experience*. *My child was respected and comfortable with the process”*
- *“The study was good*, *and a good effort was made with dairy selections”*
- *“The reminders were very helpful and reassuring*. *The rest of the study was great”*

### Effects of parental offered bribes and distraction on child compliance

Despite verbal and written information on application of the child-centred approach at assessments, 22 of 47 parents (46.8%) offered a bribe (n = 3; 6.4%), a distraction (n = 10; 21.3%) or both types of reinforcement (n = 9; 19.1%) to their child to participate in the baseline blood test. However, children who were not offered any type of parental reinforcement were more likely to be compliant with this blood test (p = 0.008). More parents offered such reinforcements at blood tests (46.8% at baseline and 35.7% post-intervention), than for the BodPod (28.6% and 13.3%, respectively), but there was lower compliance overall for the blood tests. Parents reduced the number of offered bribes and distractions at the final clinic visit.

## Discussion

### Child compliance with assessments

We investigated the feasibility and acceptability of the Milky Way Study Respectful Approach to Child-centred Healthcare (ReACH) approach, using child compliance with assessments (being willing to try on the day) as an appropriate measure of success in our study. By this definition, 95% of children were prepared to have their body composition assessed in the BodPod; 89% to have blood pressure measured, and 92% (baseline) and 87% (post-intervention) to have blood drawn at study clinics.

To enable blood draw comparisons with other studies, we need to consider rates of success rather than compliance: 77.1% of our study participants achieved a successful draw at baseline and 71.7% post-intervention. This is an excellent result, given that blood draws have been reported to fail in up to 44% of children across a variety of settings [48]. For comparison with reported ‘first attempt’ success, or a successful initial venous access on the day, we achieved rates of 70.8% at baseline and 65.2% at the final clinic, after removal of one participant per clinic who achieved a successful venous access via the second arm attempted, and two participants per clinic who achieved success at a follow-up appointment. On this basis, tertiary hospital phlebotomists achieved ‘first attempt’ success rates of 81.9% with Turkish inpatients aged 4–10 years (mean 7.2 ± 2.2 years; n = 155) [50]. In a Netherlands tertiary paediatric hospital, successful inner elbow venous access insertions, including cannulation, were reported for 78.0% of 3–11 year-olds in the operating theatre (n = 534), and for 76.9% in the outpatient unit (n = 91) [56]. However, in hospital-based studies success could be influenced by the theatre environment, including non-specialised phlebotomists and/or sedated children, while the need for treatment decisions around procedural schedules may have excluded consideration of child assent or dissent [56].

In our study, technical issues with locating suitable veins and parent-influenced child needle fear appeared the major reasons why child compliance did not always produce success, although EMLA product packaging noted a side-effect of vein-shrinkage. Our study phlebotomist reported 10 (12.2%) instances of difficult intravenous access among compliant participants, with two of these (2.4% of total compliance) achieving a successful draw from the second arm. Comparatively, market research into blood draws in children reported that 43% of children needed at least three needle sticks and 16% involved painful needle probing, or "fishing," to gain venous access [48].

In a paediatric hospital study, difficult vascular access and assessed Difficult Intravenous Access scores were found to be negatively correlated (p < 0.001 for both; n = 155) with first-attempt (on the day) venipuncture success among 4–10 year-old inpatients [50]. In findings similar to our own, this study noted that the major predictors of failure were the child’s fear, self-reported anxiety before venipuncture, and difficult vascular access. Another paediatric hospital study also appeared to support our findings, that increasing child age and phlebotomy practitioner were the major predictors of successful intravenous access [56]. However, this finding was for 3–11 year-old children in the operating theatre, where phlebotomy could be performed by a range of healthcare professionals, but not for those in outpatient care [56]. Additionally, duration of the most recent previous venipuncture procedure was found to adversely affect the success of subsequent blood draws [50]; this factor may have contributed towards our lower success rates at the final blood draws, by which time all compliant study children had attempted at least one previous blood test.

We did not allow restraint of any of our participants during blood tests. It has been estimated that 38% of 3–10 year-olds need to be physically restrained for a conventional, needle-based blood draw [48]; however, such restraint during procedures can also be proposed or initiated by the parents. In a Swedish hospital study into child participation in their own care (n = 32 children; total care procedures observed = 300), some parents were observed to lose patience, restrain their child themselves and urge the healthcare professional to proceed [25]. Nurses have described the loss of control experienced by restrained children as an ‘injustice’, potentially worse than the needle itself [23]. We believe that the compliance figures we obtained for our Milky Way Study participant blood draws without any such restraint show that a child-centred approach can form part of a medical phlebotomy protocol and achieve acceptable rates of success. Our ReACH approach included promotion of active coping behaviours in the children [45, 49, 57], with parents commenting favourably on researcher-led controlled breathing before the blood test (Box 4).

### Evaluation of how we operationalized ReACH principles into practice

Researcher evaluation of our ReACH approach, as developed and applied within the Milky Way Study, showed that assigned clinic adherence scores improved slightly from baseline to final clinics, potentially as participants and researchers become familiar and more confident with the study proceedings. Our study included healthy children in the research setting. By comparison, researchers in Sweden observed and rated active child participation in their own hospital care, including emergency care, in 32 children (18 girls; mean age 8; age range = 2–18 years). Out of 300 optimal and non-optimal care situations, assessed from level 1 (healthcare worker does not listen to child’s opinions and wishes) to level 5 (healthcare worker acts in accordance with child’s opinions and wishes), 73 cases (24.3%) were rated as optimal care at level 4, and 153 cases (51%) at level 5. This suggests that optimal active child participation in their own care is possible in the majority of hospital care procedures, but specialist paediatric healthcare professionals need to receive appropriate training in child-centred care to achieve this goal [25]. If ‘non-optimal’ care at each level reflects a relative failure of healthcare professionals to appropriately engage the child in each case, this study appears to provide support not only for child-centred care [25] in general, but also for our three foundation ReACH principles [37, 38] around child communication, concerns and participation, summarised in Box 1.

Parents reported they were satisfied with the Milky Way Study protocol and clinical assessments: 87–95% of all parental baseline and final clinic assessment evaluations met or exceeded the satisfaction cut-off. Similarly, all parents evaluated indicated they were satisfied with the Milky Way Study as a whole after the final clinic visit. Representative qualitative comments noted in Box 4 appeared to endorse our key principles and application of the ReACH approach, as summarised in Box 1. We concluded that such direct and relevant feedback appeared to highlight the value of using pre-trial qualitative research [32, 33, 58] to inform our study methodology [22], stakeholder consultation and engagement throughout [28, 31, 45], and basing all study clinical procedures on a respectful approach that promotes the fundamental rights of the child in healthcare and research [11, 34]. As we found in our community consultation, parents valued consideration of the needs and preferences of their children [22, 59].

We observed a positive association between child satisfaction and compliance frequencies, significant for the baseline BodPod clinic assessment. We both anticipated, and observed, that many children were apprehensive about blood tests prior to and during the first blood test clinic. As has been trialled successfully in other child studies [53], we added a specific category for the blood test itself to our Child Comfort Evaluation Tool, to isolate the effects of venipuncture and associated discomfort from the remaining clinic procedures. Several children gave lower evaluation scores to their blood tests, and/or to application of EMLA patches, but rated the rest of these clinics highly; for us, the ultimate positive evaluation was that they appeared happy to return for follow-up. This has important implications in the healthcare setting, as a negative experience may affect future child willingness to actively participate in the procedure, with possible withdrawal, while a positive experience can improve future motivation and engagement [25]. Discussing these problems and our various improvements with the children built trust and allowed them to become stakeholders in improving the research experience [16, 18, 28, 60].

### Parenting behaviours, needle fear and ReACH adherence

Overall, our analysis appeared to show that the majority of children were compliant when there was no bribe or distraction from the parent. We feel that this endorses further testing of the ReACH approach, and suggests that the child-centred education and advice we gave parents at baseline could be more strongly targeted in future application and investigation. Parents made fewer offers of reinforcement at the final clinic, possibly because they were becoming more accustomed to our study ReACH approach.

A variety of practical and psychological approaches have been used to help manage pain and/or associated anxiety in paediatric clinical procedures, such as psycho-education, play therapy, medical play, deep breathing and/or relaxation, comforting language, music, hypnosis and/or hypnotic suggestions of make-believe scenarios, filmed modelling of behaviours, cognitive behavioural rehearsal and therapy, and classical operant techniques such as positive and/or negative reinforcement [45, 49, 51, 57, 61]. To date, cognitive behavioural therapy and hypnosis alone have been considered to provide validated efficacy in pain management [49]. Psychological approaches used with children should promote self-control and encourage active participation in their own care [49]. However, care is recommended in dealing with accompanying parents, as parental emotions and attachment may affect the child’s coping mechanisms [57].

In our study, both distraction, known as negative reinforcement in the psychology behavioural literature, and, bribes, known as positive reinforcement [51], appeared to have a negative effect on child behaviour and to influence the child’s independent understanding of the situation. Refining application of our ReACH approach over the study has strengthened our belief that parents should avoid either type of manipulation, and the ReACH adherence tool scores both these aspects as zero. The ReACH researcher attempted to build a comfortable relationship with each child, using authentic explanations at eye level with the child to enhance trust. Crouching or kneeling to match position and establish eye contact, rather than towering over small children, has been recommended as critical in establishing trust ahead of medical procedures [57]. However well-intended, parents—possibly reacting instinctively and emotionally to a medical-type setting—could displace their child’s considered assent and trust in the healthcare professional or researcher with a distraction, by effectively removing their attention from the procedure. This could disrupt activation and enhancement of the child’s innate coping mechanisms [57], while negating both prior parental consent and child assent.

A particular issue with distraction proved to be our initial use of an iPad tablet programmed with space-themed activities. Some children became engrossed in the iPad, disrupting developing researcher engagement and trust. We consistently found that appropriate researcher explanations and continued engagement with each child filled waiting times and proved true to ReACH principles. In a rapidly progressing digital age, healthcare facilities aiming for a more child-friendly environment increasingly include digital games and touch screen devices, often presented on arrival to distract children from upcoming procedures. While agreeing that it should be a major therapeutic objective of hospitals to reduce anxiety and help children cope with the stress of upcoming procedures [45], we regard this development as an inappropriate use of negative reinforcement, and Australian ethical guidelines strongly discourage distracting children from understanding what is happening [7, 24]. We feel that our findings provide preliminary support for prioritising respectful child familiarisation, education and engagement as part of healthcare paediatric protocols; offers of digital device entertainment should be reserved for convalescence, or once the child is confident and knowledgeable about upcoming procedures. We did find the iPad useful for showing the children the study video in the initial warm-up time in the clinic visit if they had not already viewed it beforehand.

We observed several examples of negative reinforcement during blood tests, where parents did not ask but positioned smart phone videos between their child and the ReACH researcher or phlebotomist. This distracted the child, and consequently they were startled by the needle, and could jerk away and cry. Despite frequently mentioning their own fears of small spaces to us, parents made fewer attempts to distract their children from the BodPod than from blood tests. This suggests that parental needle fear may have strongly affected their attitudes and behaviours when their child had to undergo the procedure.

Child vaccinations can often result in needle fear, generally manifesting from around 5–10 years of age [21]. If not treated, the grown adult may model this fear to their children, and our community consultation revealed parent needle fear as a dominant theme [22]. A good argument has been made for consistent and appropriate pain management as the best way to break this generational cycle of needle fear [21, 47, 62], but application in the Australian healthcare sector mostly depends on parents purchasing and applying such anaesthetics. Although the use of topical anaesthetic appeared to reduce parent anxiety prior to blood collection, the 60-minute activation time seemed to heighten anxiety, and, potentially, anxiety-related pain [19] in the children. In previous research, fear and anxiety in children before venipuncture in the hospital setting was negatively correlated (p = 0.048; n = 155) with phlebotomy success [50].

We observed that parents openly well-disposed to research, and children who loved science, were the most likely to exhibit interest in the blood test instead of fear, with very high rates of compliance in these children. In our opinion, targeted parent education is needed to overcome deep-rooted needle fear, so that they can provide practical support to their children [23]. Research to improve paediatric venipuncture success rates could include onsite and/or online appropriate and respectful education of all parents and children before the first such procedure.

### Developing a holistic child-centred approach

We aimed to develop a model that placed and maintained the child at the centre of our focus; that acknowledged the parent/caregiver/family as an authority on their own child [18], and that could apply across research and healthcare settings. We developed and refined the principles and philosophy of the ReACH approach over the course of our clinical trial, with the ultimate hope of extending and embedding this work into future research and healthcare provision [35].

The widely-favoured family-centred model of care can be applied imperfectly and inconsistently by different healthcare professionals [36], particularly problematic for hospitalised children [63]. This has resulted in unintended consequences for the child, such as effectively marginalising active participation in their own care [25, 30, 34], as well as for their care-givers, such as placing barriers to involvement, communication and role negotiation [36]. The emerging framework of child-centred care aligned well with our aims, as it focuses consistently on the child at the centre of the family, and attempts to reconcile the key strengths and challenges of family-centred care by allowing for a participatory partnership between the child, their parents and paediatric healthcare professionals trained in its use [17, 25, 35, 36].

We have discussed the Bronfenbrenner ecological model [40, 41] as a suitable theoretical model for a holistic child-centred approach across a range of paediatric settings. As we found, respectful paediatric research should, ultimately, be able to ease the interactive response of the child to unfamiliar faces and unfamiliar settings [23] through planning that includes family support from the earliest stages of recruitment and study information. Additionally, combining respectful observation of the engaged child and their family with study information on a range of family and cultural settings may help provide more objective and consistent researcher evaluation, and might help illuminate why participants and parents show different behaviours in the laboratory versus their home setting [40]. Issues of child behaviour and responses being affected by researcher observation is one definition of the so-called ‘Hawthorne effect’, which has been noted across study participation and evaluation for over 100 years, and should, potentially, be able to be refined and factored into current research [64]; better understanding of family dynamics might help control for responder bias in both participant and parental evaluation.

### Informed assent and dissent

Current practice considers 7–9 year-old children to have acceptable developmental maturity to provide a satisfactory level of informed assent [8, 24, 62, 65]. However, in our experience children aged 4–7 years understood our explanations and resources, asking relevant questions and providing informed comments. We feel that our broader assessment of the emotional and developmental maturity of 4–7 year-olds is justified, at least where a consistent child-centred approach is applied and the concept of dissent is fully factored into the model [11, 13, 24]. Likewise, children as young as four years have appeared capable of sophisticated description, problem solving and decisional reasoning around treatment in a healthcare facility, when similarly provided with a secure and conducive environment [19, 60]. We consider that application of a child-centred approach, including pre-education, familiarisation, playtime and invited participation, could help formulate an appropriate and respectful level of informed assent for this age-group in research and healthcare settings.

### Study strengths and limitations

Our feasibility study appeared to show positive associations between clinic adherence to ReACH principles and child compliance with body composition, blood pressure and blood tests. Final researcher assessment of ReACH adherence was not compared across study time-points, as changes incorporated were progressive over the year. We regard our high rates of compliance for these assessments as providing preliminary support for further testing of the ReACH approach in the research setting, while noting that associations could be bidirectional and that we do not suggest any causality. Our mixed methods approach combined analysis of quantitative process evaluation data, triangulated from three different stakeholder perspectives, and qualitative responses. Further research is necessary to assess any outcome or impact evaluation in the context of each paediatric setting.

To reduce potential feedback bias by parents, we provided them with unidentified evaluation questionnaires in a plain envelope to complete, seal and return, and instructions included an altruistic appeal to help improve our study for other children, and for research in general. We found that parental qualitative evaluation at both time-points included a mix of positives and constructive criticism. After observing that early child evaluation appeared distracted and aimed to please, we engaged the child and their parent simultaneously at separate tables during evaluation. In addition to a possible ‘Hawthorne effect’ [64] on each child due to researcher observation, we also had to factor in the family dynamics of parental observation and intervention on the child’s responses, as well as sibling distractions. In strengthening child focus on evaluating each clinic procedure, not just the assessments, our Child Comfort Evaluation Tool colouring-in activity appeared to ameliorate social desirability bias and to provide thoughtful and considered answers.

There were concerns that some parents misunderstood the study ReACH principles, despite supplying them with verbal and written information at several study time-points. When they opposed the approach at critical moments, such as hurrying a child who was not ready, this was noted to compromise ReACH adherence, child comfort and even compliance. In a few cases, parents who maintained a ‘gatekeeper’ (authoritarian) stance, by making firm decisions for their child when no assent was shown, misinterpreted our respectful approach as ‘mollycoddling’. Similarly, in the Swedish hospital setting, some parents were observed to be more involved in making decisions than their child in certain situations; and could be regarded as unsympathetic when they did not request or support their child’s requests for alternative solutions to painful and unpleasant procedures [25].

The ReACH adherence tool is subjective. The ReACH researcher carried out and assessed clinic visits using the tool, while the study chief investigator performed a check of ReACH adherence ratings for a sample of children across all clinics. As the ReACH researcher was both involved in running the sessions and scoring the session with the tool, this presents a risk of bias due to a potential conflict of interest. Future studies could investigate recording sessions and subsequent coding by an independent researcher, to help control for this potential source of bias.

The study design does not include a biochemical marker to validate observations, and further research might consider using salivary cortisol as an objective indicator of child anxiety or stress. Finally, this evaluation of ReACH has only been conducted in the setting of one clinical trial. Although results are promising, further research in additional trials is warranted to justify routine inclusion of ReACH in future paediatric research.

## Conclusions

Our preliminary findings indicate that the ReACH approach is likely to be effective and acceptable, and justify further investigation in additional paediatric research. We suggest that researchers ensure that parents fully understand and accept this kind of approach prior to enrolling their children in child-centred research, and provide education around parental needle fear where required. Respecting our young participants at all times should be a core principle and a key promise across the full spectrum of child research and healthcare settings.

## Supporting information

S1 File Milky Way Study ReACH de-identified data. (XLSX)

S2 File Detailed description on incorporating emerging ReACH principles into Milky Way Study clinics and specific child assessments. (PDF)
