# Supplementary material for: Using a Respectful Approach to Child-centred Healthcare (ReACH) in a paediatric clinical trial: A feasibility study
Source: PLoS One. 2020 Nov 9;15(11):e0241764. doi: 10.1371/journal.pone.0241764 (PMC7652280; doi:10.1371/journal.pone.0241764)
Supplement: S2 File — (PDF) [file pone.0241764.s002.pdf]

S2 File:

Detailed description on incorporating emerging ReACH principles into Milky Way Study clinics and specific child assessments

As there is to date no published study protocol for the emerging Respectful Approach to Child-centred Healthcare (ReACH), this file provides fuller details than the PLoS One manuscript around incorporating ReACH principles into Milky Way Study clinics and specific child assessments. All figures cited are shown in the PLoS One manuscript; references cited here are included at the end of this document.

Materials and Methods

Clinical assessments

To allow adequate time for familiarity, we split baseline assessments over two clinic visits. We ran a single follow-up clinic, as children had already performed all the assessments at baseline and therefore did not need additional time for awareness and understanding. At the start of each clinic visit, the child and their ReACH researcher had around 15-20 minutes of 'settle-in' playtime. This allowed the ReACH researcher time for sensitive observation of the child, and allowed the child time for familiarisation with the clinic setting and the day's assessments [1]. Each clinic had a pictorial schedule of assessments, a chart with detachable photographs that could be pulled off and posted in a special post-box when each assessment was completed, as shown in **Figure 2**. (The legal guardian of the participant in this figure has given written informed consent (as outlined in PLOS consent form) to publish these details.) This acted as a timeline for the session. We considered our combination of pre-education,

playtime, familiarisation and invited participation to be a respectful and appropriate level of informed assent.

Fig 2. ReACH resource showing the sequence of tests at a trial clinic visit, with detachable photographs able to serve as a visual guide both to test procedures and to track progress over the visit: from left to right photographs present represent assessment of weight, waist measurement and BodPod body composition analysis (top row); blood pressure, strength, blood test and 'graduation' certificate awarded after all clinics completed (lower row, cut off) (Written informed consent has been provided by the legal guardian on behalf of the individual in these photographs (as outlined in PLOS consent form) to publish these case details. Fig 2 has been reprinted from [2] under a CC BY license, with permission from MDPI, original copyright 2018.)

Baseline body composition assessments: The first clinic visit involved collecting anthropometric measurements, including weight and height; neck and waist circumference, and determination of body fat mass and fat-free mass; we also determined blood pressure. Body composition was measured in the BodPod (COSMED, Rome, Italy), using non-invasive, radiation-free air displacement plethysmography to determine fat mass and fat-free (lean) mass. The study BodPod had a paediatric option (BodPod Pediatric Option™ GS model, COSMED USA Inc., San Francisco, CA), which included a seat for children less than 6 years. Children were separated from their parents inside the capsule, but as indicated in **Figure 3**, the BodPod had a large window and they were able to maintain visual and auditory contact with their parents and the ReACH researcher throughout.

Fig 3. Excerpts from the Milky Way Study Child Information Leaflet:

Upper: Astronaut training in the Bod Pod. The Bod Pod uses non-invasive air-displacement to measure body composition (lean mass and fat mass). Participants need to sit inside during testing, and there is a paediatric seat for children under six years of age
Lower: Having blood samples taken

(Pages reproduced from the Milky Way Study Child Information Leaflet. Fig 3 was previously published as two separate figures, and has been reprinted from [2] under a CC BY license, with permission from MDPI, original copyright 2018.)

Blood pressure measurements were taken in the BodPod after three to five minutes at rest, using a calibrated Dinamap ProCare 300 Monitor (GE Medical Systems, Tampa, Florida) with an appropriate paediatric cuff. We provided an authentic explanation for the blood pressure procedure, including how the cuff might feel, as shown in **Box 2**. After several children confused 'blood pressure' with having a blood test, we referred to the procedure consistently as measuring 'arm pressure'.

Box 2. ReACH-based explanations and basic strategies used in clinical assessments.

Procedure / equipment	Typical explanation
Topical anaesthetic (EMLA) cream/patches	This cream will make the skin feel numb. Numb means when you can't feel much in that spot for a while.
Cream activation time: 60 minutes	It will take about an hour to work. There are lots of toys you can choose from to play with while we wait, or games we can play.
Phlebotomy room	We can all go over to the collection room. It has everything we need in the room. Your mum/dad will come too.
Patch removal	We have a special adhesive remover so that we can remove your patch comfortably. It will take a bit longer, but we have found other children are much happier with this remover as the patch is very sticky.
Tourniquet	This is a bit like a seatbelt for your arm. It is used to help find the best veins. I will try it on both your arms today but just so that I can see which will be the best arm today. It may feel a bit tight.
**Blood collection**	I am ready to take a small amount of blood from just under the skin. The cream will have made the area numb, but you might feel some pressure. Because of the numbing cream some children say they don't feel anything at all; others say they feel a poke on their skin. Everyone is different: you can tell me how you feel it worked for you.<br>Some children like to watch, and others don't: it is up to you. Your job is to sit nice and still and breathe in through the nose and out through the mouth. This will help you be the boss of your own feelings.

Protective tape after blood collection	You have been very brave today. I just need to put a bit of pressure on the spot where the blood was collected. In a minute we can tape a little cotton wool on the spot, and you can take this off after about 15 minutes, or longer if you want. The blood will stop coming out, and your body will fix up the hole quickly.
**Blood pressure cuff and procedure**	This is an arm pressure [*as opposed to 'blood pressure'*] cuff; it goes around your arm and gets quite tight. It is a bit like a floatie [*a flotation device for both arms, used in swimming pools to help keep young children afloat*], where the air blows into the floatie and (it) hugs around your arm. It doesn't stay tight for long, and we can watch how the numbers go down on this monitor as it slowly returns to normal.<br>The arm pressure tells us how strongly your heart is pumping blood to your arm.
**BodPod body composition assessment**	The BodPod, or rocket ship, measures what your body is made of. There are a couple of things you need to know about our rocket ship, or BodPod. There is a window that you can see us through, and we can see you. I will be watching, and you will hear me talking to you, letting you know how much time there is left. If at any time you want me to open the door, you can place your hands on your head, like this, and I will open the door. I would like you to sit nice and still inside the rocket ship, and we will close the door for one minute and then open it to see how you are. This will happen 3 times. You will hear a whooshing noise - sounds like a "whoosh, whoosh" [*similar to a bicycle pump pumping up a tyre*] - and you will also hear a clunking noise - like a "clunk…… clunk". These sounds mean that the machine is working.

After assessments, a choice of breakfast items was offered to all children, and the participant chose a small thank-you gift from a selection of items. The child was not aware of the thank you gift beforehand, to avoid this acting as an undue incentive. While the children ate breakfast, the lead researcher explained sample and data collection, including our sociodemographic questionnaire, to the parent.

Baseline blood test: The second baseline clinic visit required a fasting blood test. Application of anaesthetic patches is recommended to prevent child needle fear [1, 3, 4], and this was endorsed by parents at our pre-trial community consultation [2]. However, we considered it respectful to give children an informed choice. EMLA (AstraZeneca, Sweden) numbing

patches were optimally applied to both inner arms, to allow the phlebotomist a choice of the best venipuncture site. Package instructions and our own in-house tests established that a 50-60-minute wait was needed for local cutaneous absorption and optimal numbness. A potential disadvantage was that the EMLA active ingredients, lidocaine and prilocaine, may have a side-effect of vein-shrinkage. We preferred to supervise the application procedure onsite, rather than sending items home with parents to apply before the visit. This allowed us to measure time of application accurately, and to ensure that patches were placed correctly.

After children complained about painful removals of the anaesthetic patches, we added an adhesive-removal solvent wipe to the process (Smith & Nephew REMOVE Adhesive remover, St-Laurent, Canada. Ingredients: isoparaffin, dipropylene glycol methyl ether, aloe extract, benzyl alcohol, fragrance). This took about 2-3 minutes per arm, in which time some children showed signs of anxiety. To address this, the ReACH researcher and the phlebotomist engaged the children by making a fun ‘race’ of this process, each taking a patch off one arm at the same time. The EMLA anaesthetic patches were largely effective at eliminating pain, but not feelings of touch or needle insertion. We made this distinction clear to the children and their parents prior to the test [1].

We employed an experienced paediatric phlebotomist, following our community consultation finding that most parents considered this the best guarantee of a good child experience [2]. By minimising both physical and psychological harm, and keeping the child and their parent informed and involved in the moment, we hoped that the children would feel they were doing something worthwhile even if it meant some discomfort [5]. The phlebotomist offered children the opportunity to help with setting up the different coloured blood collection vials in the tray. Children were also given a choice of squeezy toy to assist in vein location. We

developed and applied a ReACH confident momentum strategy to empower children who expressed fear about the test: we acknowledged their feelings and reassured them that the ReACH researcher had a strategy previously used to help other study children, placing them in charge of their own feelings. Our ReACH strategy for blood tests hence incorporated a combination of pharmacological and psychological approaches, ongoing monitoring of the participant and invited participation of the child and their parent in the way we implemented the blood test [6].

Immediately before the procedure, the ReACH researcher provided an age-appropriate, authentic explanation of the procedure and what the child's role would be. Children were not held down, as was the current accepted practice in paediatric phlebotomy at the time of writing [1, 7], but asked to do a task involving sitting still and breathing 'in through the nose and out through the mouth'. Our pictorial Child Information Leaflet represented the child sitting alone, but we gave children the option of sitting with their parent if they preferred (as described in Figure 3). Our deep-breathing technique, described as 'helping you be the boss of your own feelings' promoted relaxation and awareness, to make the situation less stressful for both parent and child.

Final body composition and blood test assessments: The third clinic, held after three months of dairy intervention, incorporated all previous assessments. As per baseline procedures, we sent text and email reminders to help prepare the child and their family, and reviewed the day's procedures at the start of the clinic [8], using the pictorial schedule shown in Figure 2. At the end of the clinic, children 'graduated' from our Milky Way Study 'Astronaut Training' with a personal participation certificate, and parents could take a space-themed astronaut

photograph as a memento. Themed progress reports with appropriate assessment results were mailed to each child at a later date.

Additional ReACH strategies and tools

Enablers and barriers: Parents offering their children incentives to participate in assessments is ethically dubious. It may cause some children to believe there is something to fear about doing the assessments, thereby increasing the child's anxiety and reducing the likelihood they will complete the assessment. Paediatric healthcare workers should be trained to recognise verbal and non-verbal dissent [5], and researchers, in particular, should not continue if the child remains clearly unwilling after a suitable pause to explore their understanding of the procedure [9]. Practical incorporation of ReACH principles into child assessments made us expand our ideas of incentives as 'bribes' or 'distractions', concepts often used interchangeably in the literature [1, 9], to incorporate the behavioural concepts of positive and negative reinforcement [10]. These were investigated for impact on our ReACH-based assessments.

We defined a bribe as a parent offering a form of positive reinforcement, such as a treat or reward for later (delayed gratification). Similarly, a distraction was defined as a parent offering any form of negative reinforcement that could break eye-contact with the researcher, trust and/or session momentum. Our ReACH approach was designed with the belief that distracting a child before they are fully ready for a procedure can result in a sudden awareness, such as finding a needle in their arm without warning, which could negate choice, validation of the experience and informed assent.

Study confident momentum tools included the age-appropriate explanations and basic strategies shown in Box 2. An iPad mini electronic touch screen tablet, programmed with space-themed and educational games, was initially used with the first 7% of children to undergo baseline assessments, particularly in the BodPod as the rocket ship ‘on-board computer’, to help keep children relaxed during the assessment [2, 11]. However, this method frequently proved a negative reinforcement, or a barrier, as it distracted the child’s focus from the ReACH researcher and assessment explanations. Use of the iPad was therefore phased out.

ReACH Adherence Tool: The ReACH researcher assessed adherence to the child-centred approach during each clinic visit. Seven key principles were used to develop the tool’s adherence categories for our study: mutual trust, respect, sensitive observation, quality care-giving, a prepared environment, time for uninterrupted play and consistency [1, 12]. The ReACH Adherence Tool comprises 13 questions, as shown in **Box 3.** Application enabled the ReACH researcher to maintain a consistent clinical schedule, while tailoring each clinic around the specific characteristics of the child [13].

Box 3. ReACH Adherence Tool for the child researcher to evaluate adherence to ReACH principles and the study protocol during each clinic visit. A score of 13 implies very high adherence.

A Respectful Approach to Child-centred Healthcare (ReACH) Adherence Checklist

For application by ReACH Researcher at each child-centred clinic visit

TRUST

1. **Researcher’s trust in child’s competence to complete session using ReACH approach only** Confident (1) Little/ no trust (0)
   Parent and child anxiety levels gauged on arrival and acknowledged by providing more time for introductions / play / session information as required

2. **Distraction from parent** None (1) ≥ 1 distraction (0)
   E.g. 'Don't look' when child is distressed/ offers video clip on mobile phone / holds child's head to look away
3. **Bribe from parent** None (1) ≥ 1 distraction (0)
   *E.g. If you do this 'I'll take you to (*restaurant*)' / 'I'll buy you...'*
4. **Distraction from researcher** None (1) ≥ 1 distraction (0)

AUTHENTICITY (Communication with child is honest, upfront and genuine)

5. **Study information/ video shown to child before assessments** Yes (1) No (0)
   Procedures explained via video / easy and engaging visual tools
6. **Researcher honest about potential discomfort** Yes (1) No (0)
   Explore visual and sensory explanations, e.g. 'Some children say they feel some pressure, others say they don't feel anything at all. Would you like me to show you on my arm?' [If yes, Researcher applies pressure with fingernail onto own arm.] *'You can try it on your arm, and tell me how it feels'*
7. **Researcher uses authentic language and tone** Yes (1) No (0)
   Speaks authentically and explains procedures using simple photographic/ visual devices and open discussion (no baby talk, forced games or other placating examples)
8. **Protocol went according to plan, as initially described to child** Yes (1) No (0)
   Where protocol deviates, Researcher stops, acknowledges and explains the discrepancy before seeking child's approval to continue

OBSERVATION (Sensitive observation for insight into child's needs)

9. **Researcher focus** Yes (1) No (0)
   Researcher watches for and acknowledges distress cues, particularly when the child looks to the Researcher during explanations and procedures; maintains eye contact throughout procedures where has promised to do so
10. **Researcher finds insights into child's personality, to reach them on their level and make connection(s)** Yes (1) No (0)
    During play time each child can form a unique connection with the Researcher. This develops and strengthens as the Researcher applies the ReACH approach, particularly if the child is interested in the research

ENVIRONMENT

11. **Safe to express feelings** Feelings not investigated (0) Feelings investigated / acknowledged (1)
12. **Playtime allowed** <15 mins (0) ≥15 min (1)
13. **Control of play** (child instigates) Researcher led (0) Child led (1)

Child Comfort Evaluation Tool: The ReACH researcher developed a customised, laminated evaluation tool for each clinic visit, to assess child comfort (or satisfaction) with clinic procedures and assessments. This was derived from a combination of two tools validated for pre-assessment of dental anxiety in young children, the 7-faces Revised Smiley Faces Program, for computer application in children aged 4-11 years [14], and the recommended alternative for paper-based assessment, the 5-faces version of the Modified Child Dental Anxiety Scale, for children aged over five years [15]. Assessment and interpretation of pain in young children have traditionally been regarded as difficult [3], and there is as yet no validated scale for children less than six years to report acute pain, such as needle pain [16]. The study Child Comfort Evaluation Tool gave each child the opportunity to identify the appropriate face on a 5-faces Likert smiley-face assessment scale, placed opposite a colour photograph of each procedure in that clinic, as shown in **Figure 4** (the legal guardian of the participant in this figure has given written informed consent (as outlined in PLOS consent form) to publish these details).

Fig 4. Child Comfort Evaluation Tool for self-evaluation of comfort and satisfaction with Milky Way Study clinic sessions and assessments. This figure is similar but not identical to the original image used in the study and is provided for illustrative purposes only.
(Written informed consent has been provided by the legal guardian on behalf of the individual in these photographs (as outlined in PLOS consent form) to publish these case details)

References

1. Karlsson K, Rydstrom I, Enskar K, Englund AC. Nurses' perspectives on supporting children during needle-related medical procedures. Int J Qual Stud Health Well-being. 2014;9:23063. doi: 10.3402/qhw.v9.23063. PubMed PMID: 24646473; PubMed Central PMCID: PMCPMC3955765.
2. Nicholl A, O'Sullivan TA. Keep calm and carry on: parental opinions on improving clinical dietary trials for young children. Nutrients. 2018;10(9). doi: 10.3390/nu10091166. PubMed PMID: 30149614; PubMed Central PMCID: PMCPMC6163458.
3. Melhuish S, Payne H. Nurses' attitudes to pain management during routine venepuncture in young children. Paediatr Nurs. 2006;18(2):20-3. PubMed PMID: 16544799.
4. McMurtry CM, Pillai Riddell R, Taddio A, Racine N, Asmundson GJG, Noel M, et al. Far from "just a poke": common painful needle procedures and the development of needle fear. Clin J Pain. 2015;31:S3-S11. doi: 10.1097/ajp.0000000000000272. PubMed PMID: 00002508-201510001-00003.
5. McIntosh N, Bates P, Brykczynska G, Dunstan G, Goldman A, Harvey D, et al. Guidelines for the ethical conduct of medical research involving children. Royal College of Paediatrics and Child Health: Ethics Advisory Committee. Arch Dis Child. 2000;82(2):177-82. doi: 10.1136/adc.82.2.177.
6. International Association for the Study of Pain [IASP]. 2019 global year against pain in the most vulnerable: fact sheets on pain in the most vulnerable Washington DC: IASP; 2019 [cited 2019, June 9]. Available from: www.iasp-pain.org/GlobalYear?navItemNumber=580.

7. Behm S, Dunn T. Blood draws: a mandate to improve patient experience [Medical Technology & Innovation]. Online: Beckers Hospital Review; 2016 [updated February 10, 2016; cited 2019 March 8]. Available from: https://www.beckershospitalreview.com/quality/blood-draws-a-mandate-to-improve-patient-experience.html.

8. Gerçeker GÖ, Ayar D, Özdemir EZ, Bektaş M. The impact of the difficult vascular access, fear, and anxiety level in children on the success of first-time phlebotomy. J Vasc Access. 2018;19(6):620-5. doi: 10.1177/1129729818765598. PubMed PMID: 29562830.

9. Spriggs M. Understanding consent in research involving children: the ethical issues (a handbook for human research ethics committees and researchers). Melbourne: University of Melbourne; 2010 [cited 2019, February 2]. Available from: https://scholar.google.com/scholar_lookup?hl=en&publication_year=2010&author=M+Spriggs&title=Understanding+Consent+in+Research+Involving+Children%3A+The+Ethical+Issues.

10. Passer MW, Smith RE. Psychology: the science of mind and behaviour. Australian ed. North Ryde, NSW.: McGraw-Hill Australia; 2013.

11. Marechal C, Berthiller J, Tosetti S, Cogniat B, Desombres H, Bouvet L, et al. Children and parental anxiolysis in paediatric ambulatory surgery: a randomized controlled study comparing 0.3 mg kg−1 midazolam to tablet computer based interactive distraction. Br J Anaesth. 2017;118(2):247-53. doi: 10.1093/bja/aew436.

12. Marlen D. All about…: Pikler. Nursery World. 2017;5:23-7. doi: 10.12968/nuwa.2017.5.23.

13. Capurso M, Ragni B. Psycho-educational preparation of children for anaesthesia: A review of intervention methods. Patient Educ Couns 2016;99(2):173-85. doi: https://doi.org/10.1016/j.pec.2015.09.004.

14. Buchanan H. Assessing dental anxiety in children: the Revised Smiley Faces Program. Child Care Health Dev. 2010;36(4):534-8. doi: 10.1111/j.1365-2214.2009.01033.x. PubMed PMID: 20184594.
15. Howard KE, Freeman R. Reliability and validity of a faces version of the Modified Child Dental Anxiety Scale. Int J Paediatr Dent. 2007;17(4):281-8. Epub 2007/06/15. doi: 10.1111/j.1365-263X.2007.00830.x. PubMed PMID: 17559456.
16. Birnie KA, Hundert AS, Lalloo C, Nguyen C, Stinson JN. Recommendations for selection of self-report pain intensity measures in children and adolescents: a systematic review and quality assessment of measurement properties. Pain. 2019;160(1):5-18. Epub 2018/09/05. doi: 10.1097/j.pain.0000000000001377. PubMed PMID: 30180088.
